# The genome and gene editing system of sea barleygrass provide a novel platform for cereal domestication and stress tolerance studies

**DOI:** 10.1016/j.xplc.2022.100333

**Published:** 2022-05-05

**Authors:** Liuhui Kuang, Qiufang Shen, Liyang Chen, Lingzhen Ye, Tao Yan, Zhong-Hua Chen, Robbie Waugh, Qi Li, Lu Huang, Shengguan Cai, Liangbo Fu, Pengwei Xing, Kai Wang, Jiari Shao, Feibo Wu, Lixi Jiang, Dezhi Wu, Guoping Zhang

**Affiliations:** 1 Department of Agronomy, Key Laboratory of Crop Germplasm Resource of Zhejiang Province, Zhejiang University, Hangzhou 310058, China; 2 Novogene Bioinformatics Institute, Beijing 100083, China; 3 College of Agronomy, Hunan Agricultural University, Changsha 410128, China; 4 School of Science, Hawkesbury Institute for the Environment, Western Sydney University, Richmond, NSW 2753, Australia; 5 The James Hutton Institute, Dundee DD2 5DA, UK; 6 The Division of Plant Sciences, School of Life Sciences, University of Dundee, Dundee DD2 5DA, UK; 7 School of Agriculture and Wine & Waite Research Institute, University of Adelaide, Waite Campus, Glen Osmond, SA 5064, Australia

**Keywords:** sea barleygrass, salt tolerance, genome, transcriptome, divergence

## Abstract

The tribe Triticeae provides important staple cereal crops and contains elite wild species with wide genetic diversity and high tolerance to abiotic stresses. Sea barleygrass (*Hordeum marinum* Huds.), a wild Triticeae species, thrives in saline marshlands and is well known for its high tolerance to salinity and waterlogging. Here, a 3.82-Gb high-quality reference genome of sea barleygrass is assembled *de novo*, with 3.69 Gb (96.8%) of its sequences anchored onto seven chromosomes. In total, 41 045 high-confidence (HC) genes are annotated by homology, *de novo* prediction, and transcriptome analysis. Phylogenetics, non-synonymous/synonymous mutation ratios (Ka/Ks), and transcriptomic and functional analyses provide genetic evidence for the divergence in morphology and salt tolerance among sea barleygrass, barley, and wheat. The large variation in post-domestication genes (e.g. *IPA1* and *MOC1*) may cause interspecies differences in plant morphology. The extremely high salt tolerance of sea barleygrass is mainly attributed to low Na^+^ uptake and root-to-shoot translocation, which are mainly controlled by SOS1, HKT, and NHX transporters. *Agrobacterium*-mediated transformation and CRISPR/Cas9-mediated gene editing systems were developed for sea barleygrass to promote its utilization for exploration and functional studies of hub genes and for the genetic improvement of cereal crops.

## Teaser

A genome assembly and gene editing system for the wild Triticeae species sea barleygrass provide a powerful platform for gene functional research and genetic improvement of cereals.

## Introduction

Major cereal crops in the grass family (Poaceae) diverged around 10 million years ago (mya). However, many crop-related grasses have remained recalcitrant to domestication. Only a few of them, particularly those with large grains, adapted to cultivation and became staple crops for human civilization (IRGSP, 2005; Murphy, 2007; Schnable et al., 2009; Mascher et al., 2017; IWGSC, 2018). Triticeae is one of the most economically important tribes in the grass family, containing many domesticated crops, including wheat (*Triticum aestivum*), barley (*Hordeum vulgare*), and rye (*Secale cereal*) (Feuillet and Salse, 2009). Globally, approximately 900 million tons of Triticeae crops are produced annually, accounting for ∼30% of the total cereal production (FAOSTAT, http://faostat.fao.org/) (FAO, 2020). In addition, a large number of wild Triticeae species exhibit extensive genetic variation, distinct morphological traits, and high tolerance to a range of environmental stresses. Therefore, more attention has been paid to these wild relatives of cereal crops for the development of crop cultivars with high yield potential and excellent abiotic and biotic stress tolerance (Nevo and Chen, 2010; Avni et al., 2017; Wang et al., 2018).

Triticeae crops emerged in the Fertile Crescent of the Near East and diverged from oats (*Avena sativa* L.) around 25 mya (Gaut, 2002). The original ancestors of the wheat and barley lineages diverged around 13 mya (Gaut, 2002). During their subsequent evolution, they independently experienced dramatic divergence in both morphology and environmental stress adaptation (Glemin and Bataillon, 2009). For example, integration of the D genome from *Aegilops tauschii* into tetraploid wheat *Triticum turgidum* improves both environmental adaptation and grain quality of common wheat (Jia et al., 2013). The *TmHKT1;5-A* gene from *Triticum monococcum* significantly increases the grain yield of durum wheat by 25% on saline soils (Munns et al., 2012). The introgression of *Fhb7* from *Thinopyrum elongatum* confers resistance to both fusarium head blight and crown rot in diverse wheat backgrounds without yield penalty (Wang et al., 2020). During domestication, some features of wild grasses, such as excessive tillers and brittle rachis (seed shattering), were weakened, and some important agronomic characteristics, including moderate plant height and large spike and seed size, were eventually retained through selection and breeding (Wang et al., 2019). Meanwhile, the cultivated Triticeae species show wide variation in responses to abiotic stresses, such as cold tolerance in rye (Bauer et al., 2017), aluminum tolerance in wheat (Delhaize et al., 2004), and salt tolerance in barley (Munns and Tester, 2008; Russell et al., 2016). However, in comparison with their domesticated crop descendants, wild Triticeae species exhibit even wider genetic diversity and higher tolerance to abiotic stresses (Maccaferri et al., 2009; Munns et al., 2012; Wang et al., 2020).

Sea barleygrass (*Hordeum marinum* Huds.), an annual halophyte in salt marshes, consists of two subspecies, *marinum* (2*n* = 2*x* = 14) and *gussoneanum* (2*n* = 2*x* = 14 or 2*n* = 4*x* = 28), and is characterized by the distinctive Xa genome (Jakob et al., 2007; Carmona et al., 2013). The diploid *marinum* is distributed throughout the Mediterranean countries, and the tetraploid *gussoneanum* overlaps with the diploids only in the far eastern Mediterranean region, expanding into Asia (Jakob et al., 2007). Sea barleygrass is well known for its extremely high salt and waterlogging tolerance and stronger tillering ability compared with Triticeae crops including barley and wheat; it is considered one of the major genetic sources of salt tolerance in cereal crop improvement (Garthwaite et al., 2005; Malik et al., 2011; Alamri et al., 2013; Huang et al., 2018). In fact, the obtained amphidiploid wheat hybrids with sea barleygrass (ssp. *marinum*) show a much higher salt tolerance than wheat (Islam et al., 2007). Also, overexpression of *HKT1;5* from *H. marinum* produces higher salt tolerance in transgenic rice than the ortholog from *H. vulgare* (Huang et al., 2019). However, little progress has been made in research on abiotic stress tolerance, evolutionary genetics, comparative genomics, and the use of sea barleygrass in crop genetic improvement because of the lack of a high-quality genome assembly and efficient transformation system. Recently, the genomes of barley, wheat, and their progenitors have been successively sequenced (Avni et al., 2017; Luo et al., 2017; IWGSC, 2018; Mascher et al., 2021; Zhu et al., 2021), laying the foundation for deciphering the genome of sea barleygrass. Accordingly, we first completed a reference genome sequence of the diploid sea barleygrass (*H. marinum* ssp. *marinum*) accession H559 using the combined technologies of Illumina, PacBio single-molecule real-time (SMRT) sequencing, 10x Genomics, and high-throughput chromosome conformation capture (Hi-C). In this study, we developed an efficient transformation and CRISPR/Cas9-mediated genome editing system for sea barleygrass. Moreover, integrative genomic, transcriptomic, and functional analyses were performed to clarify the molecular mechanisms underlying the differences in morphology and salt tolerance among sea barleygrass, barley, and wheat.

## Results and discussion

### *De novo* assembly and annotation of the *H. marinum* genome

The genome size of *H. marinum* accession H559 was estimated to be 4.2 Gb by flow cytometry (supplemental Figure 1), smaller than that of barley cv. Morex (Mascher et al., 2017), which is consistent with a previous study (Jakob et al., 2004). Furthermore, the H559 genome was estimated to be 3996 Mb based on *K*-mer analysis of the 266.7-Gb Illumina HiSeq (2 × 150 bp) dataset (supplemental Table 1). The *de novo* genome assembly was constructed using a combination of Illumina HiSeq (789.1 Gb), PacBio SMRT (325.3 Gb), 10x Genomics (388.4 Gb), and Hi-C (434.9 Gb) data, which achieved about 510× coverage of the H559 genome (supplemental Figure 2A and supplemental Table 2). The average fragment length and N50 of the reads in the PacBio library were 9.38 and 15.72 kb, respectively. The assembled genome contains a total length of 3816 Mb (95.5% of the estimated genome by *K*-mer analysis), which is approximately 684 Mb smaller than that of the *H. vulgare* assembly (Mascher et al., 2021), with a contig N50 size of 6.83 Mb and a contig N90 size of 1.81 Mb (Table 1). There were only 0.21 Mb of N bases in the gap regions (Gap N) of the sea barleygrass genome, fewer than those of barley (1.33 Mb) and wheat (75.26 Mb) (Mascher et al., 2021; Zhu et al., 2021).

**Table 1:** Statistics and composition of the sea barleygrass genome

Assembly statistics	Values
Estimate of genome size (Mb)	3996
Total length of scaffolds (Mb)	3816
Total number of scaffolds	1197
Scaffold N50 (Mb)	524.47
Scaffold N90 (Mb)	450.13
Total number of contigs	2090
Contig N50 (Mb)	6.83
Contig N90 (Mb)	1.81
Gap counts	893
Gap length (Mb)	0.09
Anchored to the pseudo-chromosomes (Mb)	3694
GC content (%)	44.5
Percentage of repeat sequences	3137 Mb (82.2%)
High-confidence (HC) genes	41 045
Low-confidence (LC) genes	38 822
Complete BUSCOs (%)	98.4

To evaluate the accuracy of the genome assembly, small-fragment library reads were compared with the assembled genome using BWA software (http://bio-bwa.sourceforge.net/), resulting in an alignment ratio of 99.8% (supplemental Table 3). Furthermore, the Hi-C-assisted assembly using LACHESIS (https://github.com/shendurelab/LACHESIS) compiled 1197 assembled scaffolds, anchoring a total of 3694 Mb of sequences onto seven pseudo-chromosomes. This represents 96.8% of the assembled *H. marinum* genome, with each chromosome ranging between 450 and 588 Mb in length (Table 1, supplemental Table 4, and supplemental Figure 2B). According to BUSCO analysis (https://busco.ezlab.org/), 98.4% of all Embryophyta core genes were detected in the *H. marinum* assembly, similar to the wheat (99.0%) and barley (98.4%) reference genomes (supplemental Table 5).

Similar to the sequenced genomes of other Triticeae species (Bauer et al., 2017; Mascher et al., 2021; Zhu et al., 2021), the sea barleygrass genome contains abundant repeat sequences. The high quality of the *H. marinum* genome assembly was further validated by assessment of long terminal repeat (LTR) completeness using the LTR Assembly Index (LAI = 12.7) (supplemental Table 5) (Ou et al., 2018). A total of 3137 Mb of sequences (82.2% of the genome) were annotated as transposable elements (TEs) (Table 1 and supplemental Table 6). The LTR retrotransposons (LTR-RTs, e.g. Copia and Gypsy) were the major components, accounting for 67.2% of the genome, followed by 11.2% DNA transposons. For instance, there are 1.38 Gb of Gypsy retrotransposons in the genome of sea barleygrass, whereas there are 2.08 Gb of Gypsy retrotransposons in the genome of cultivated barley (supplemental Table 6). The Gypsy retrotransposons *Cereba* and *Quinta* are enriched in centromeric regions in *Triticum* species (Li et al., 2013). Based on the density distribution of *Cereba* and *Quinta*, we obtained the centromere positions of the seven chromosomes of sea barleygrass (supplemental Figure 3 and supplemental Table 7). The average length of the centromeric region for each chromosome was 7.16 Mb (supplemental Table 7).

**Figure 1:**
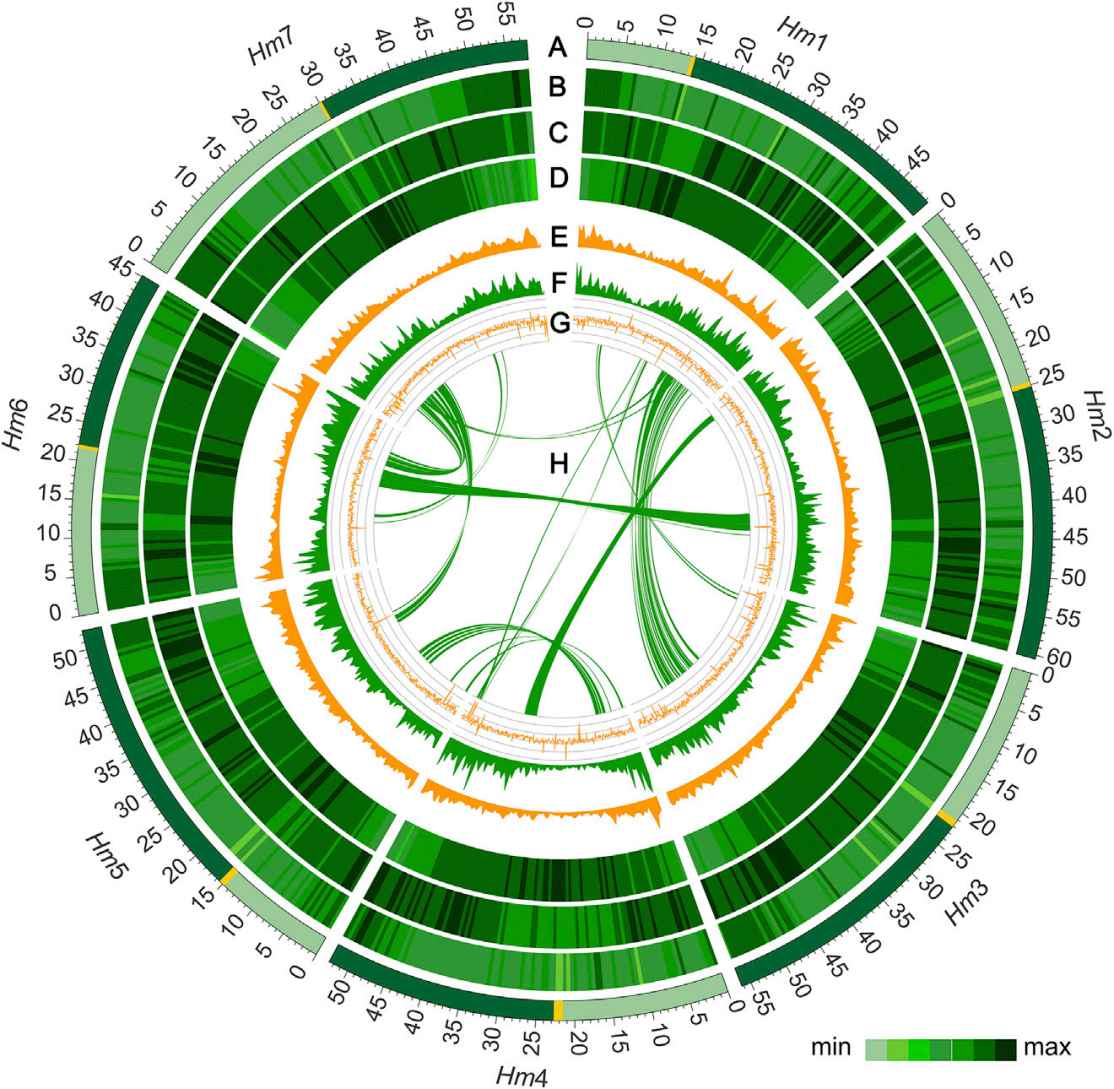
Circular diagram showing features of the genome of sea barleygrass accession H559. **(A)** Chromosome name and size; tick size indicates 10 Mb, and yellow regions indicate centromeres. Deep and light colors represent the long and short arms of seven chromosomes. **(B)** Density of DNA transposons. **(C)** Density of LTR-Copia. **(D)** Density of LTR-Gypsy. **(E)** Density of genes. **(F)** Expression levels of genes in the RNA-seq experiment. **(G)** GC content (%). **(H)** Homologous relationships of chromosomes.

There were 41 045 high-confidence (HC) and 38 822 low-confidence (LC) genes annotated by homology, *de novo* prediction, and transcriptome analysis (RNA-seq data from roots, leaves, stems, spikes, and developing grains) (Table 1). Overall, 97.4% of the HC genes were anchored onto the seven chromosomes, and 88.4% were assigned predicted functions according to protein databases (supplemental Tables 4 and 8). The average exon number per gene (4.1 exons) of *H. marinum* was similar to that of *H. vulgare* (4.3 exons), *Zea mays* (4.1 exons), and *Sorghum bicolor* (4.3 exons) (Paterson et al., 2009; Schnable et al., 2009) but less than that of *T. aestivum* (∼5.3 exons, AABBDD) (supplemental Table 9) and slightly more than that of *Oryza sativa* (3.8 exons) (IRGSP, 2005). Moreover, 19 941 miRNAs, 1335 tRNAs, 2497 rRNAs, and 921 snRNAs were annotated in the genome (supplemental Table 10). The genomic features of the seven chromosomes are shown in Figure 1.

### Comparative analysis of *H. marinum* and other plant genomes

**Figure 2:**
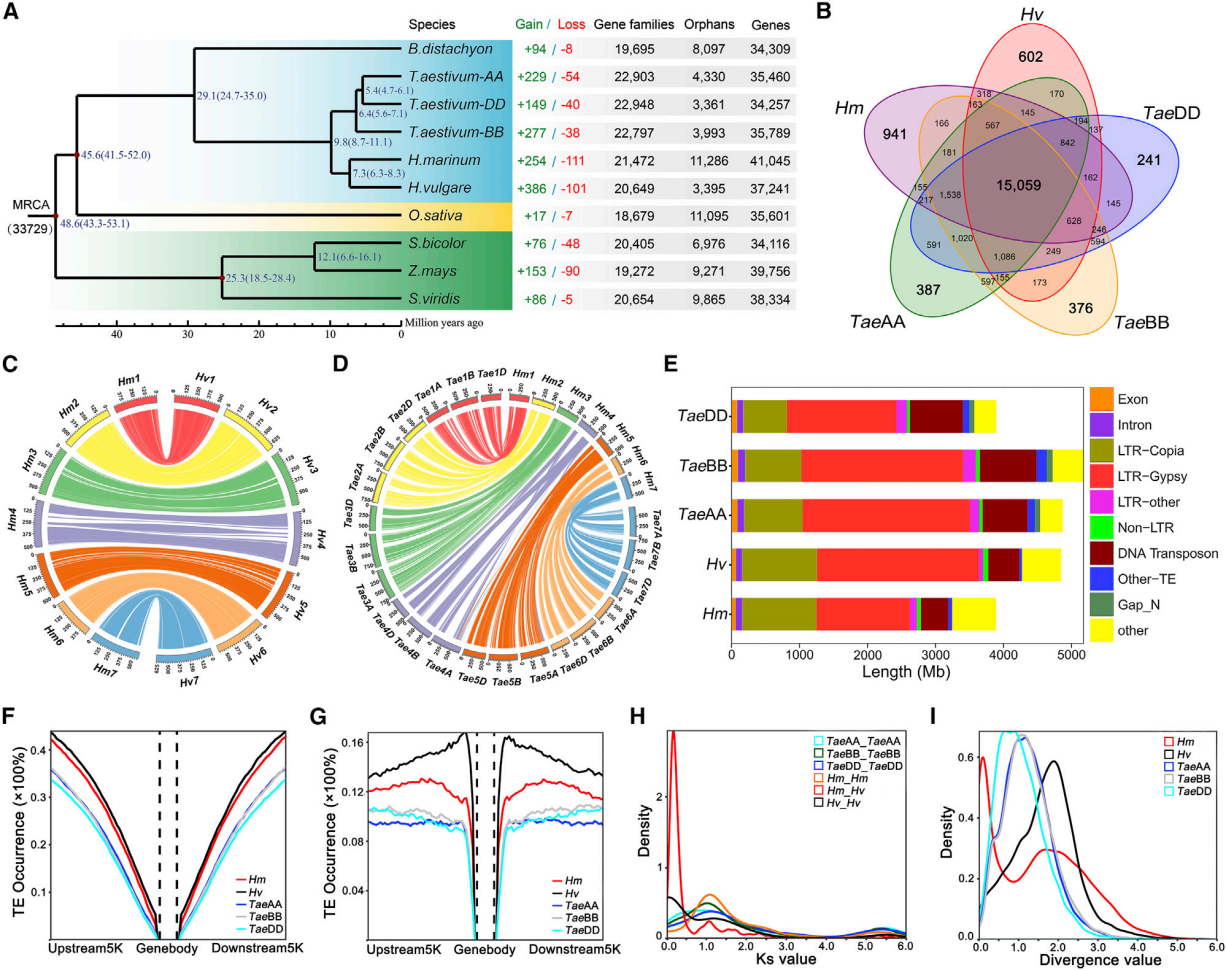
Genomic features in the Triticeae tribe. **(A)** Phylogenetic tree, divergence time estimates, and gene family gain (+)/loss (−) among the genomes of *O. sativa*, *S. bicolor*, *Z. mays*, *S. viridis*, *B. distachyon*, *H. vulgare*, *T. aestivum* (AA, BB, and DD subgenomes), and *H. marinum*. The numbers of gene families and orphans (unclustered gene families) and the number of annotated genes are indicated next to each genome. **(B)** Venn diagram of common and unique gene families in the genomes of sea barleygrass, barley, and wheat. **(C and D)** Collinearity of seven chromosomes between sea barleygrass and barley **(C)** and between sea barleygrass and wheat **(D)**. **(E)** Comparison of the genomic components in sea barleygrass, barley, and wheat. **(F and G)** The occurrence of LTR retrotransposons **(F)** and DNA transposons **(G)** in the upstream and downstream (∼5 kb) gene regions in the genomes of sea barleygrass, barley, and wheat. Different colored lines indicate the different genomes. **(H)** WGD analysis based on Ks values. **(I)** The estimated insertion times of LTRs in the genomes of sea barleygrass, barley, and wheat. The estimated insertion time (mya) was calculated by T = K/2r (r = 1.3 × 10^−8^) and corrected with the JC69 model.

Comparative genomics and evolutionary analysis were performed on eight grass genome assemblies, including those of *O. sativa*, *S. bicolor*, *Z. mays*, *Setaria viridis*, *Brachypodium distachyon*, *H. vulgare*, *T. aestivum* (AA/BB/DD), and *H. marinum* (supplemental Table 11). The number of gene families in these species was 33 731, including 4640 homologous single-copy genes. In total, 21 472 gene families were detected in the genome of *H. marinum*, and 83.12% of them (17 848) were single copy, similar to those in three *T. aestivum* subgenomes (∼82.5%) but more than those in *H. vulgare* (78.5%) and *Z. mays* (69.2%) (Figure 2A and supplemental Table 12). A phylogenetic tree constructed using the protein sequences of all homologous single-copy genes showed that sea barleygrass was most closely related to barley, followed by wheat and *B. distachyon* (Figure 2A). Using MCMCtree (Yang, 2007), the divergence times of sea barleygrass from barley and wheat were estimated at 6.3–8.3 and 8.7–11.1 mya, respectively (Figure 2A), demonstrating the closer phylogenetic relationship between sea barleygrass and *Hordeum* species (Jakob et al., 2007; Carmona et al., 2013). Among the five Triticeae genomes/subgenomes, the number of annotated genes was largest in *H. marinum* (Figure 2A). We therefore compared the structural features of genes in the sea barleygrass genome with those of barley and wheat. The average length of exons in *H. marinum* was shorter than that in *H. vulgare*, and the average exon number per gene was less than that in *T. aestivum*. Consequently, the average CDS length in *H. marinum* was 134–300 bp shorter than those in *H. vulgare* and *T. aestivum* (supplemental Figure 4 and supplemental Table 9).

In Triticeae species, 15 059 shared gene families were detected in the genomes of sea barleygrass, barley, and wheat. Based on gene ontology (GO) enrichment analysis, the 941 unique gene families in the sea barleygrass genome were mainly associated with “metabolic processes” (Figure 2B, supplemental Figure 5B, and supplemental Dataset 1). Compared with the genome of barley, 254 and 111 gene families in the sea barleygrass genome exhibited expansion and contraction, respectively (Figure 2A and supplemental Dataset 1). Interestingly, the markedly expanded gene families were in the categories of “integral component of membrane,” “oxidoreductase activity,” “electron carrier activity,” “photosynthesis,” “hydrogen ion transmembrane transporter activity,” and “proline biosynthetic process” (supplemental Figure 6A). It has been reported that expansions of *cupin* and *cytochrome P450* (*CYP*) gene families in the hornwort (*Anthoceros angustus*) genome are involved in adaptation to drought and oxidative stresses in terrestrial environments (Zhang et al., 2020a, 2020b). Hence, we assume that the expanded gene families in *H. marinum* are likely to be associated with tolerance to environmental stress. The contracted gene families were mainly involved in “anion binding,” “cellular metabolic process,” “ATP binding,” and “phosphorylation” (supplemental Figure 6B). All seven chromosomes in the genomes of sea barleygrass, barley, and wheat show high collinearity (Figure 2C and 2D, and supplemental Figure 7), except for the 4A/5A translocation region in the wheat AA subgenome (Ling et al., 2013). The genome size of sea barleygrass is similar to that of the DD subgenome of wheat (or *Aegilops tauschii*) (Jia et al., 2013; Luo et al., 2017), and its genome components are similar to those of barley except for the Gypsy retrotransposons (Figure 2E). LTR-RTs have been reported to cause genomic instability and expansion (Kaessmann et al., 2009). For instance, at least 5%–18% of plant nucleotide-binding and leucine-rich repeat proteins emerged by LTR-RT-driven retroduplication (Kim et al., 2017). Here, the expanded 0.7 Gb Gypsy retrotransposons are probably associated with 386 expanded gene families in *H. vulgare*; in comparison, 254 expanded gene families are involved in *H. marinum* (Figure 2A).

In view of the dramatic differences in TEs (LTR-RTs and DNA transposons) among sea barleygrass, barley, and wheat, we compared the distribution frequency of TEs in the upstream, downstream, and gene-body regions of the conserved genes. The distribution frequency of TEs in the upstream and downstream regions was much higher than that in the gene-body region (Figure 2F and 2G, and supplemental Figure 8). LTR-RTs and DNA transposons were most pronounced in barley, followed by sea barleygrass and wheat (Figure 2F and 2G). It was reported that TE distribution could be associated with differences in gene expression levels among plant genomes (Slotkin and Martienssen, 2007; Hollister et al., 2011; Bennetzen and Wang, 2014). Thus, the difference in TE occurrence may partially account for the differential regulation of orthologous genes among Triticeae species.

Whole genome duplication (WGD) has been a major driver of genome evolution and divergence in cereals (Mascher et al., 2017; IWGSC, 2018). Paralogous genes in sea barleygrass, barley, and wheat were identified using all-against-all BLASTP, and the Ks values of each gene pair were determined using KaKs_Calculator 2.0 to detect divergence events between different species (Wang et al., 2010). There was a peak at Ks of around 0.2, which represents a species differentiation event between sea barleygrass and barley that occurred after the α and β WGD events, with Ks values of around 1.05 and 5.5, respectively (Figure 2H). Furthermore, massive TE amplification events may affect gene transcription and generate genome evolution (Bennetzen and Wang, 2014). Considering the differences in LTR-RTs in the genomes of sea barleygrass, barley, and wheat (Figure 2E and supplemental Table 6), we estimated the insertion dates of LTR-RTs in these genomes. Activation period analyses indicated that a burst of TE activity occurred at 1.5–2.0 mya for the barley genome, 1.0–1.25 mya for the wheat AA and BB subgenomes, and 0.5–1.0 mya for the wheat DD subgenome. Interestingly, a more recent activation period (∼0.1 mya) was also found in the sea barleygrass genome, in addition to those in barley and other cereal crop genomes (Figure 2I and supplemental Figure 9).

### Divergence in plant morphology and salt tolerance among sea barleygrass, barley, and wheat

As a wild Triticeae species, sea barleygrass is, in general, morphologically and developmentally different from barley and wheat, and it is characterized by shorter plant height, stronger tillering ability, a brittle rachis, and smaller seeds (supplemental Figure 10A and 10B). For morphological divergence, we compared genetic variation in amino acid similarity, phylogeny, and Ka/Ks values of genes involved in the regulation of morphological and developmental divergence among the three plant species (supplemental Figure 10C and 10D, supplemental Table 13, and supplemental Dataset 3). On the whole, the protein sequences of these genes are quite similar in the three Triticeae species (supplemental Figure 10C). However, unlike genes associated with plant height and tillering development, genes involved in the regulation of spike and seed morphology showed relatively lower amino acid sequence similarity and larger genetic variation in sea barleygrass relative to barley and wheat according to the gene phylogeny and Ka/Ks analysis (supplemental Figure 10C and 10D and supplemental Table 13). The GRAS family protein MONOCULM 1 (MOC1) has been reported to initiate axillary buds and promote tiller development in rice (Li et al., 2003). Strigolactone (SL) plays an essential role in shoot branching. The SL receptor D14 (Dwarf 14) interacts with D3, and the D14/D3 complex then mediates D53 degradation by ubiquitination (Yao et al., 2016; Shabek et al., 2018). D53 physically interacts with or suppresses the expression of *Ideal Plant Architecture1* (*IPA1*), a key transcription factor for tillering (Song et al., 2017). A unique deleterious mutation site (Ser403Phe) at the C-terminal region of *HmIPA1* was identified in sea barleygrass after comparing it with barley and wheat (supplemental Figure 11). Transcriptomic studies using 1-month-old seedlings showed that there were higher expression levels of *MOC1*, *D14*, *D3*, and *IPA1* and lower expression level of *D53* in the shoots of H559 than in the shoots of the barley cultivar Morex and the wheat cultivar Chinese Spring (CS) (supplemental Figures 10A and 12). Thus, stronger tillering ability in H559 relative to Morex and CS could be attributed to higher expression of *IPA1* and *MOC1*. Single amino acid substitutions in HvBRI1 (BR-insensitive 1) or HvSLN1 (Slender1) were reported to cause semi-dwarf or dwarf mutants in barley (Chandler et al., 2002; Chono et al., 2003). Currently, there are two deleterious substitutions, Leu778Gln and Glu786Asp, in HmBRI1 (supplemental Figure 13) and one substitution, Pro446Ser, in HmSLN1 (supplemental Table 14). Moreover, the divergence of the grain shattering-related CTD phosphatase sh-h and the two BEL1-type homeobox proteins qSH1 and SH5 in sea barleygrass occurred earlier than those in barley and wheat, as indicated by the phylogenetic analysis (supplemental Figure 10D). The grain-size-related calmodulin-binding protein GW5 (Liu et al., 2017) and the indole-3-acetic acid-glucose hydrolase TGW6 (Ishimaru et al., 2013) showed dramatically lower Ka/Ks values in wheat and barley than in sea barleygrass (supplemental Table 13), indicating that they were positively selected in barley and wheat during domestication. Therefore, it can be assumed that differences in the structure and/or expression of these well-characterized regulators result in the divergence in plant morphology among these Triticeae species.

**Figure 3:**
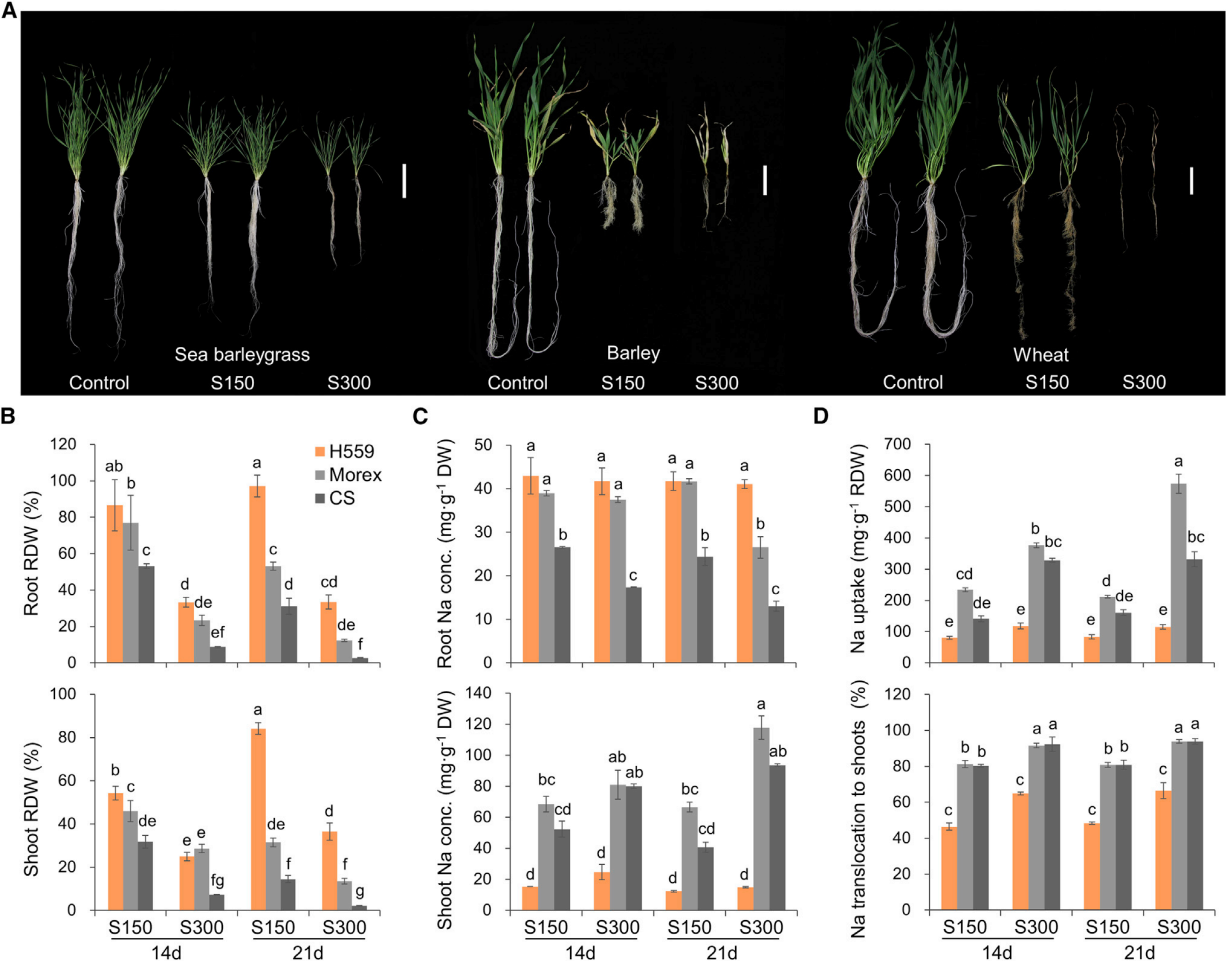
Differences in salt tolerance among H559, Morex, and Chinese Spring. **(A)** Plant growth of sea barleygrass accession H559, barley cultivar Morex, and wheat cultivar Chinese Spring (CS) after 21 days of 0 (Control), 150 (S150), and 300 (S300) mM NaCl treatments. Scale bar corresponds to 10 cm. **(B)** Relative dry weight (treatment/control) of roots and shoots. **(C)** Na^+^ concentrations in roots and shoots. **(D)** Na^+^ uptake by roots (total Na^+^ content/root dry weight) and Na^+^ translocation ratio to the shoots (%) in H559, Morex, and CS after 14 or 21 days of 150 and 300 mM salt treatments. Data are shown as mean ± SD (*n* = 6). Different small letters indicate a significant difference (*P* < 0.05) using Tukey’s test after a one-way ANOVA. CS, Chinese Spring; RDW, relative dry weight.

To confirm the extremely high salt tolerance of the halophyte sea barleygrass, we examined the growth of accession H559 exposed to 0–500 mM NaCl. As expected, H559 plants still survived under 500 mM NaCl and maintained a low Na^+^ concentration in shoots (supplemental Figure 13). We then compared the salt tolerance of sea barleygrass (H559), barley (Morex), and wheat (CS). When exposed to 150 and 300 mM NaCl for 21 days, the relative shoot DW of H559 was 2.7- and 5.8-fold greater than that of Morex and CS, respectively (Figure 3A and 3B). On the other hand, the shoot Na^+^ concentration in H559 was only 12.7%–30.5% that of Morex and 15.9%–30.9% that of CS (Figure 3C). The lower shoot Na^+^ concentration in H559 can be attributed to lower root Na^+^ uptake and root-shoot translocation (Figure 3D), confirming previous findings (Garthwaite et al., 2005; Huang et al., 2018). Under 300 mM NaCl, K^+^ concentrations in roots and shoots of H559 were much higher than those of Morex and CS (supplemental Figure 14), and H559 consequently showed a higher tissue K^+^/Na^+^ ratio. A higher K^+^/Na^+^ ratio is generally considered to be a key indicator of salt tolerance (Chen et al., 2005). In short, the high salt tolerance of sea barleygrass is related to its lower root Na^+^ uptake and root-shoot translocation and its higher K^+^ uptake.

To gain a deeper understanding of the molecular mechanisms of high salt tolerance in sea barleygrass, genes potentially involved in salt tolerance were identified and compared among the sea barleygrass, barley, and wheat genomes (supplemental Table 15). Ka/Ks analyses of orthologous genes were performed using the branch model of PAML v4.9 (Yang, 2007). The higher Ka/Ks values of genes involved in Na^+^/K^+^ homeostasis, response to oxidative stress, and ABA signaling and synthesis in sea barleygrass suggested that they were under stronger evolutionary selection than those in barley and wheat (supplemental Table 15). This finding is consistent with the results obtained from GO enrichment analysis of the expanded gene families in the sea barleygrass genome, which highlighted the roles of “integral component of membrane,” “hydrogen ion transmembrane transporter activity,” and “oxidoreductase activity” (supplemental Figure 6A).

**Figure 4:**
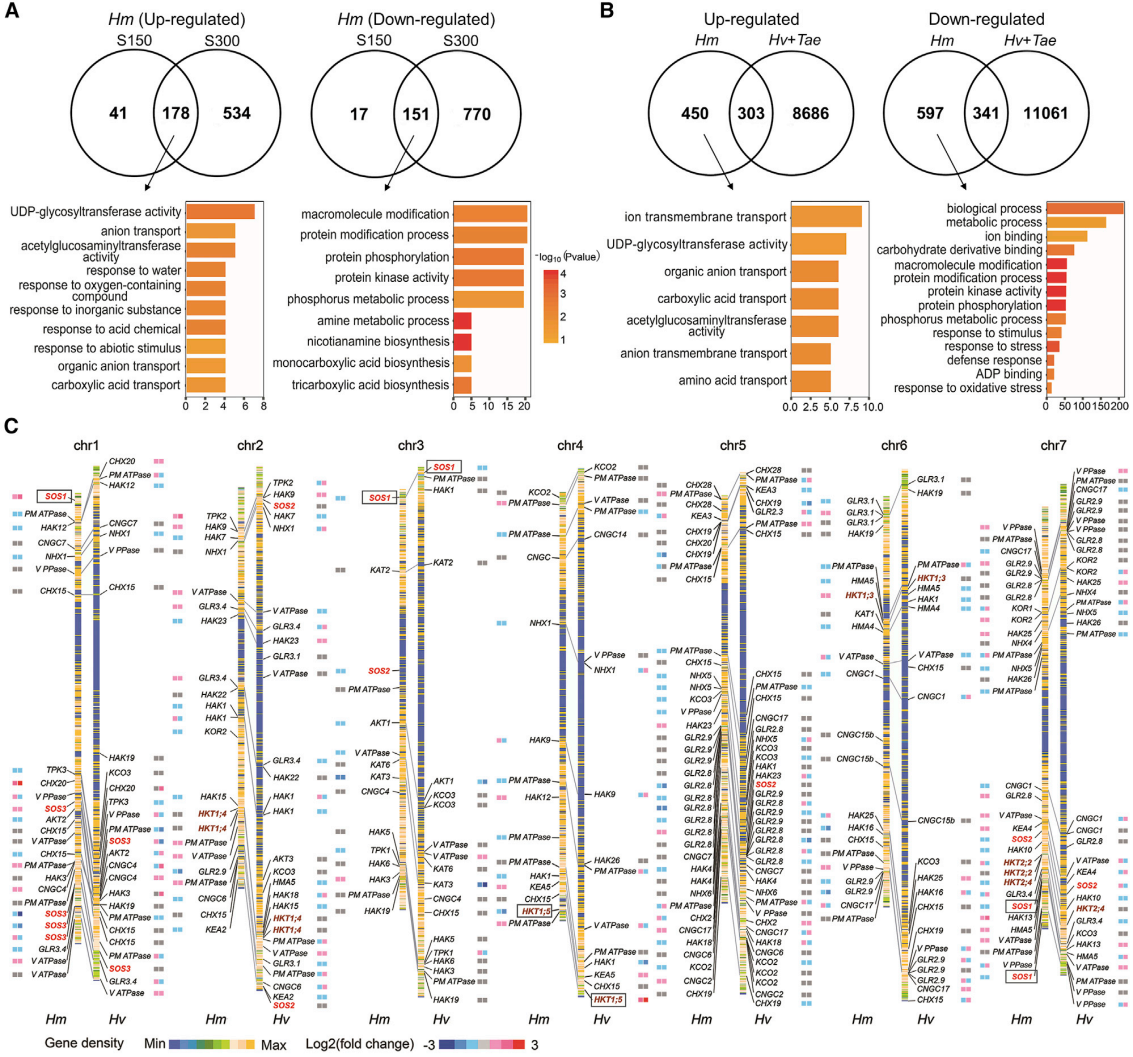
Transcriptomic analysis among H559, Morex, and Chinese Spring in response to salt treatments. **(A)** Differentially expressed genes (DEGs) (|log_2_fold change| ≥ 2, FDR < 0.05) and GO enrichment in the roots of H559 after 4 days of 150 and 300 mM salt treatments. **(B)** DEGs and GO enrichment in the roots between H559 and Morex/CS. **(C)** Chromosome position and heatmap of fold changes of homologous genes related to ion homeostasis in the roots of sea barleygrass and barley after 4 days of 150 (S150) and 300 (S300) mM salt treatments.

To identify differentially expressed genes (DEGs) in response to salt stress in H559, Morex, and CS, RNA-seq analysis was performed using RNA isolated from roots and shoots of plants exposed to 0, 150, and 300 mM NaCl for 4 days (Figure 4, supplemental Figures 15 and 16, and supplemental Datasets 4 and 5). The correlations (R^2^) between the RNA-seq and the qRT–PCR data were 0.91 (H559), 0.87 (Morex), and 0.89 (CS), respectively (supplemental Figure 17). In three Triticeae species, numerous genes (DEGs, |log_2_fold change| ≥ 2, FDR < 0.05) involved in ion homeostasis, antioxidant activity, and ABA signaling and synthesis showed a salt-induced response (supplemental Figure 18), consistent with the results obtained from the Ka/Ks analysis (supplemental Table 16). In total, 219 and 712 DEGs were upregulated in the roots of H559 after 4 days of exposure to 150 and 300 mM NaCl, respectively. Of these, 178 upregulated DEGs associated with “UDP-glycosyltransferase activity,” “anion transport,” and “response to abiotic stimulus” were shared in roots of H559 exposed to the two NaCl treatments, whereas 168 and 921 DEGs were downregulated under the 150 and 300 mM NaCl treatments, respectively (Figure 4A). Compared with Morex and CS, H559 had 450 upregulated and 597 downregulated unique DEGs (Figure 4B). The upregulated DEGs were mainly involved in “ion transmembrane transport” and “UDP-glycosyltransferase activity,” whereas the downregulated DEGs were mainly involved in “ion binding” and “metabolic processes” (Figure 4B). Most interestingly, these pathways were consistent with the GO enrichment of expanded and contracted gene families in the sea barleygrass genome (supplemental Figure 6). Sea barleygrass may therefore have adapted to saline environments through unique salt-adaptive gene family expansion/contraction during evolution (Munns, 2011). KEGG enrichment analysis of these DEGs in H559 revealed that “phenylpropanoid biosynthesis” and “phenylalanine metabolism” pathways were dramatically repressed and that “flavonoid biosynthesis” was significantly enhanced (supplemental Figure 19). Flavonoids, secondary metabolites of the phenylalanine metabolism pathway, would likely accumulate under various abiotic stresses to reduce oxidative stress (Nakabayashi et al., 2014). Meanwhile, glycosylation catalyzed by glycosyltransferases plays an important role in the stability and biological activity of flavonoids (Le Roy et al., 2016). Hence, we suggest that the increased UDP-glycosyltransferase activity in sea barleygrass might be responsible for the redirection of metabolic flux to flavonoid biosynthesis (Dong et al., 2020) to accumulate flavonoid glycosides for survival under high salinity (supplemental Figure 20). In addition, 406 DEGs showed deleterious amino acid variants, and the roles of “metabolic process,” “kinase activity,” and “transporter activity” were highlighted in sea barleygrass accession H559 for its adaptation to saline environments (supplemental Figure 21).

We further examined the salt stress-induced expression of DEGs associated with ion homeostasis in H559, Morex, and CS, focusing on the genes that regulate root Na^+^ uptake, root-shoot translocation, and tissue K^+^/Na^+^ ratio (Figure 4C, supplemental Figures 15 and 16, and supplemental Dataset 3). Salt Overly Sensitive 1 (SOS1) is a plasma membrane Na^+^/H^+^ antiporter for Na^+^ extrusion out of plant root cells (Shi et al., 2000). *SOS1* was significantly upregulated in the roots of H559 but showed little change in Morex and CS (Figure 4C and supplemental Figure 16A). Moreover, its copy number in H559 was greater than that in Morex (Figure 4C and supplemental Figure 16B). HKT sub-family 1 transporters mediate Na^+^ transport in the root vasculature and Na^+^ accumulation in shoots (Ren et al., 2005). Here, four HKT1 transporters were identified in H559 (Figure 4C and supplemental Figure 22). *High-Affinity Potassium Transporter 1;5* (*HKT1;5*) has been reported to negatively regulate salt tolerance by facilitating Na^+^ root-shoot translocation in barley, whereas its homologous genes in rice (*OsHKT1;5*) and bread wheat (*TaHKT1;5*) positively regulate their salt tolerance (Byrt et al., 2014; Kobayashi et al., 2017; Huang et al., 2020). Interestingly, *HmHKT1;5* in sea barleygrass, like *HvHKT1;5*, also showed negative regulation of salt tolerance (Huang et al., 2019). In this study, *HmHKT1;5* was more obviously downregulated in H559 than was *HvHKT1;5* in Morex under salt stress, as indicated by the relative and absolute qRT–PCR assay (Figure 4C and supplemental Figure 16E–16G). It is therefore possible that the higher salt tolerance of barley and sea barleygrass relative to wheat and rice is closely associated with the distinct difference in the pattern of salt tolerance regulation among these homologous *HKT1;5* genes. Moreover, Na^+^/H^+^ Exchangers (NHXs) and Cation/H^+^ Exchangers (CHXs) are associated with K^+^ homeostasis under stress conditions (Pardo et al., 2006; van Zelm et al., 2020). The Ka/Ks value of *NHX6* in sea barleygrass was greater than that in barley and wheat (supplemental Table 16), and *CHX20* was upregulated in roots and shoots of H559 under salt stress (Figure 4C and supplemental Figures 15C and 16A). It is likely that the differential expression of functional ion transporters, such as SOS1, HKT1;5, and CHX20, may have enhanced Na^+^ exclusion in roots and restricted Na^+^ translocation to shoots in sea barleygrass, resulting in higher K^+^/Na^+^ ratios and salt tolerance compared with barley and wheat (supplemental Figure 20).

### *Agrobacterium*-mediated transformation and CRISPR/Cas9-mediated genome editing systems for sea barleygrass

**Figure 5:**
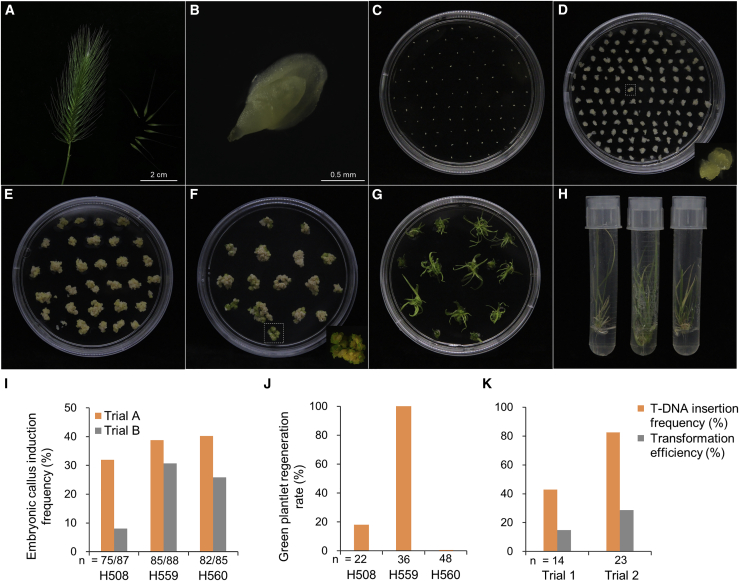
*Agrobacterium*-mediated transformation of immature sea barleygrass embryos. **(A)** Collection of immature seeds from spikes. **(B)** Isolation of immature embryos. **(C)** Immature embryo cultivation. **(D)** Embryonic callus initiation after 2-week induction. **(E)** Callus induction for 3 weeks on selection medium after *Agrobacterium* inoculation and co-cultivation. **(F)** Transformed callus starting to green and produce small shoots after 10 days under low-light conditions. **(G)** Regeneration of strong shoots. **(H)** Transgenic sea barleygrass plantlet transferred to test tube showing strong roots in hygromycin-containing medium. **(I)** Embryonic callus induction frequency of immature embryos from the three sea barleygrass accessions H508, H559, and H560. The immature seeds in Trials A and B were collected from natural and controlled-environment conditions, respectively. **(J)** Green plantlet regeneration rate of the immature-embryo-derived calli from three accessions after 1 month of cultivation. **(K)** T-DNA insertion frequency and transformation efficiency of the transformation system. The transition and regeneration media with different hygromycin concentrations were used in Trial 1 (10 mg l^−1^) and Trial 2 (20 mg l^−1^). The transformation efficiency corresponds to the product of callus induction frequency, green plantlet regeneration rate, and T-DNA insertion frequency.

We developed an efficient transformation system to more deeply explore and understand the functional genes in sea barleygrass (Figure 5A–5H). The three spp. *marinum* accessions H508, H559, and H560 were used as immature embryo donors to assess their capacity for callus induction and differentiation. After a 1-month treatment under 300 mM NaCl, the root and shoot dry weights of these accessions were 75.7%–94.1% and 54.2%–65.3% that of the control (without NaCl addition), indicating their extremely high salt tolerance (supplemental Figure 23). After a 1-month callus induction, the immature embryos of the three accessions planted under natural conditions (Trial A) showed an embryonic callus induction frequency of 32.0%–40.2%. However, the callus induction frequency was only 8.0%–30.7% when the three accessions were planted in a growth chamber (Trial B), indicating that natural field conditions were more favorable for callus induction of sea barleygrass. The immature embryos of H559 and H560 exhibited a more stable dedifferentiation activity than those of H508 (Figure 5I). The immature-embryo-derived calli were then transferred to the transition medium and produced green plantlets after 1 month of cultivation. Unlike the medium used for calli of barley and wheat (Bartlett et al., 2008; Hayta et al., 2019), the regeneration medium for regenerating plantlets of sea barleygrass required high levels of kinetin (5 mg/l). Interestingly, the green plantlet regeneration rate of accession H559 was 100% (36/36), much higher than that of H508 (4/22) and H560 (0/48) (Figure 5J). Consequently, H559 was selected as the donor of immature embryos for development of the genetic transformation system.

**Figure 6:**
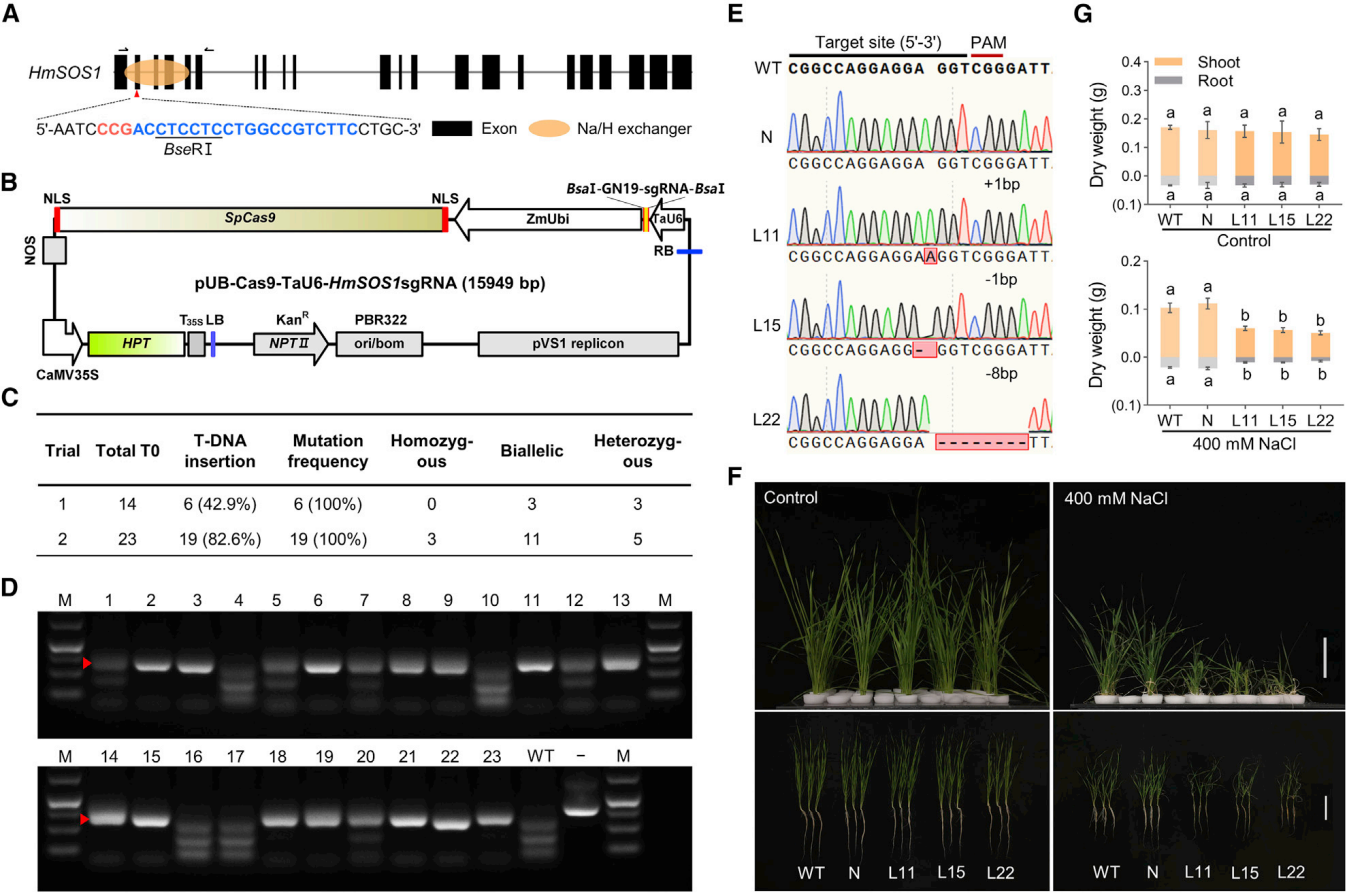
An efficient CRISPR/Cas9-mediated genome editing system for sea barleygrass. **(A)** Gene structure and sgRNA target of the *HmSOS1* gene. One sgRNA is designed to target the *HmSOS1* gene. The sgRNA target is highlighted in blue letters; red letters indicate PAM sequences, and black arrows indicate the primers used for PCR amplification. Underlined sequences are the recognition sites for *Bse*RⅠ. **(B)** Schematic model of the pUB-Cas9-TaU6-*HmSOS1*sgRNA vector. **(C)** Mutation frequency and editing efficiency of the genome editing system for sea barleygrass. The biallelic, heterozygous, and homozygous mutations were identified with the program DSDecode (http://skl.scau.edu.cn/dsdecode/). **(D)** Agarose gel showing mutations of *HmSOS1* in transgenic sea barleygrass plants detected by a PCR/RE assay. Red triangles indicate mutated bands in single-allelic and biallelic mutants. Lanes 1–23, representative PCR products of transformed plants digested with *Bse*RⅠ; WT, wild type; –, undigested PCR amplification. **(E)** Representative Sanger sequencing chromatograms for lines L11, L15, and L22 of *hmsos1* mutants. **(F and G)** Inserted and deleted bases are labeled in red boxes. Growth performance **(F)** and dry weights **(G)** of the roots (gray) and shoots (brown) of the *hmsos1* mutants and the WT (H559) after 14 days under control and salt conditions. Three-week-old seedlings were transferred to hydroponic culture supplemented with 0 (Control) and 400 mM NaCl. N, negative transgenic lines without T-DNA insertion. Values are shown as means ± SD (*n* = 6). Different small letters indicate a significant difference (*P* < 0.05) using Tukey’s test after a one-way ANOVA.

The specific guide RNA (gRNA) target site for the *HmSOS1* gene was cloned into a CRISPR/Cas9 vector in which *Cas9* was driven by the maize *Ubi* promoter (Figure 6A and 6B). The regenerated plants were obtained by the transformation of sea barleygrass cells with the target recombinant vector. In Trial 1, only 42.9% (6/14) of the plantlets showed the T-DNA insertion in the genome. When the hygromycin concentration was increased from 10 to 20 mg l^−1^ in transition and regeneration media, the T-DNA insertion frequency in Trial 2 increased to 82.6% (19/23) (Figure 5K and supplemental Figure 24). Hence, the *Agrobacterium*-mediated transformation system developed for spp. *marinum* accession H559 had a high transformation efficiency, an average of 28.7%, slightly higher than that of the barley cultivar ‘Golden Promise’ and the wheat cultivar ‘Fielder’ (Bartlett et al., 2008; Hayta et al., 2019).

Subsequently, we detected mutations in the target site region in the 25 transformed plants (6 lines in Trial 1 and 19 lines in Trial 2). The editing efficiency was 100% in the two independent trials, and 68% of the plants contained biallelic and homozygous mutations, as shown by a PCR/restriction enzyme (RE) assay and Sanger sequencing (Figure 6C and 6D). *hmsos1-11* (L11) harbored one 1-bp insertion at 241 bp downstream of the ATG, resulting in a premature stop codon. In *hmsos1-15* (L15) and *hmsos1-22* (L22), 1- and 8-bp deletions were detected at 242 and 234 bp downstream of the ATG, generating a frameshift mutation and a premature stop codon, respectively (Figure 6E). After 14 days of 400 mM salt treatment, three *hmsos1* mutant lines had much lower plant dry weights and higher shoot Na^+^ concentrations than the wild plant H559 (Figure 6F, 6G, and supplemental Figure 25), indicating that salt tolerance of these mutants was reduced compared with H559. These results suggested that the CRISPR/Cas9-mediated gene editing system for sea barleygrass was successfully developed and that the highly expressed *SOS1* gene plays a crucial role in the salt tolerance of sea barleygrass. The efficient CRISPR/Cas9-mediated genome editing system for the wild Triticeae species sea barleygrass may provide a powerful tool for gene function and genetic improvement studies in cereals.

## Materials and methods

### Plant materials

The diploid sea barleygrass accession H559 (*H. marinum* ssp. *marinum*, obtained from NordGen, Nordic Genetic Resource Center, Sweden) was used for genome sequencing and *de novo* assembly. The barley cultivar Morex and the wheat cultivar CS (CS42, kindly provided by Prof. Xiue Wang, Nanjing Agricultural University, China) were also used.

### Illumina and PacBio sequencing

High-quality genomic DNA (gDNA) was extracted from the 1-month-old plant using a modified phenol-chloroform method (Mascher et al., 2017). The sequencing libraries were constructed following the Illumina TruSeq Nano DNA Library Prep Kit user guide. Illumina sequencing libraries were finally sequenced on the Illumina HiSeq-4000 platform (paired-end, PE 150 bp). Libraries for PacBio SMRT genome sequencing were constructed following the standard protocols of Pacific Biosciences using SMRTbell Template Prep Kits. Single-molecule sequencing was performed using the PacBio Sequel System, which yielded a total of 325.3 Gb of data with 34 684 998 clean reads.

### 10x Genomics and Hi-C library construction

The method for the 10x Genomics library construction was described previously (Zheng et al., 2016). Here, we prepared the library following the Chromium Genome Reagent Kit protocol (v2 • Rev B). We then constructed Hi-C libraries using the same 1-month-old sea barleygrass seedling as input. The preparation of the biotin-labeled Hi-C samples, enriched using streptavidin C1 magnetic beads, was performed following the standard protocol (Belton et al., 2012). After end repair, A-tailing, adaptor ligation, and amplification, paired-end sequencing was performed on the Illumina HiSeq-2500 platform.

### *De novo* assembly and assessment

The genome assembly was performed based on a protocol described previously (Zhang et al., 2020a, 2020b). Illumina, PacBio, 10x Genomics, and Hi-C data were combined to optimize the accuracy of sea barleygrass genome assembly (supplemental Figure 2A). Before assembling, “daligner” executed by the main script of the FALCON assembler (https://github.com/PacificBiosciences/FALCON) was used to correct PacBio long reads to generate consensus sequences. After error correction, the consensus sequences achieved accuracies up to 99.999%. Then, FALCON identified the overlaps between all pairs of preassembled error-corrected reads, which were used to construct a directed string graph. Contigs were constructed by finding the paths from the string graph. Error correction of the preceding assembly was performed with the consensus-calling algorithm Quiver (https://manpages.ubuntu.com/manpages/xenial/man1/quiver.1). Base-pair correction of the assembly was performed using Pilon (https://github.com/broadinstitute/pilon). Linked reads generated from 10x Genomics were aligned to the consensus sequence of the PacBio assembly to obtain the superScaffold using Bowtie 2 (http://bowtie-bio.sourceforge.net/bowtie2). fragScaff software (https://sourceforge.net/projects/fragscaff/) was mainly used for 10x Genomics scaffold extension. The resulting contigs or scaffolds were finally anchored and oriented onto seven pseudo-chromosomes by Hi-C. First, clean Hi-C paired-end reads from the Illumina platform were aligned to the draft assembly, and repeat and junk reads were filtered out to obtain high-quality data. Then reads close to the restriction sites were extracted for chromosome-scale scaffolding of the *de novo* reference assembly, and the manual correction was finally processed using Juicebox (https://github.com/aidenlab/Juicebox).

To assess the completeness of the assembled *H. marinum* genome, we performed BUSCO analysis by searching against the Embryophyta universal benchmarking single-copy orthologs (BUSCOs, version 3.0). We also assessed the completeness of the LTR-RTs in the *H. marinum* genome by LAI (Ou et al., 2018).

### Genome size estimation

The flow cytometry analysis of H559 genome size was performed using leaves from 2-week-old seedlings of H559, Morex, and CS. The cell nucleus suspension was prepared with Otto buffer-1 (100 mmol/l citric acid, 1% [v/v] Tween 20 [pH 2.3]) and analyzed in a FACSCalibur Flow Cytometer (Becton Dickinson, USA). A total of 266.7 Gb of Illumina HiSeq data were used to perform genome size estimation by *K*-mer analysis. The genome size was estimated using the formula: Genome size = *K*-mer_Number/Peak_Depth.

### RNA isolation and transcriptome sequencing

Total RNA was isolated from different tissues (root, leaf, stem, spike, and developing grains) of sea barleygrass H559 using TRIzol reagent (Invitrogen, CA, USA). RNA purity and integrity were assessed using a NanoPhotometer spectrophotometer (IMPLEN, CA, USA) and a Bioanalyzer 2100 system (Agilent Technologies, CA, USA) based on RIN > 7.0. RNA concentration was measured using the Qubit RNA Assay Kit on a Qubit 2.0 Fluorometer (Life Technologies, CA, USA). Approximately 3 μg of high-quality RNA sample was used for sequencing library preparation according to a previous study (Sun et al., 2020). The 125-bp paired-end sequencing was performed on the Illumina HiSeq 2500 platform (Illumina, San Diego, USA). The transcriptome sequencing data were finally polished for genomic gene prediction and annotation.

### Annotation of repetitive sequences and non-coding RNAs

For repeat annotation, we performed a combination of homologous sequence alignment and *de novo* prediction. First, we searched for repetitive sequences that were similar to known repeats in the Triticeae repeat sequence database (https://github.com/jdaron/CLARI-TE) using RepeatMasker and RepeatProteinMask (http://www.repeatmasker.org/). We identified tandem repeats in the *H. marinum* genome using Tandem Repeats Finder (http://tandem.bu.edu/trf/trf.html). To determine the centromeric regions, we obtained the Gypsy family RLG_famc8.3 (*Cereba*) and RLG_famc8.1/8.2 (*Quinta*) annotation information from the TE annotation results, and we calculated their density distribution across each chromosome of H559. The annotations for miRNAs, tRNAs, rRNAs, snRNAs, and other non-coding RNAs in the *H. marinum* genome were based on a previously reported method (IWGSC, 2018).

### Gene prediction and annotation

For gene structure prediction, we combined homology-based prediction, *de novo* prediction, and other evidence-supported prediction (Sun et al., 2020). We selected *H. vulgare*, *O. sativa*, *B. distachyon*, *T. aestivum*, *T. urartu*, and *A. tauschii* as homologous species of sea barleygrass. Finally, the obtained annotation results were adjusted using PASA (https://github.com/PASApipeline/PASApipeline/wiki) combined with transcriptome assembly data, and information on untranslated regions and alternative splicing was attached. The final gene set was obtained by data screening based on the presence of expression, overlap with transposons (TE), and whether only *de novo* evidence supported a given gene. The assignment of gene confidence classification was divided into two steps using criteria and methods described previously (Avni et al., 2017). First, BLASTP software (NCBI; https://www.ebi.ac.uk/Tools/sss/ncbiblast/) was used to construct an alignment of the predicted peptide sequences against known protein datasets (*Hvu*.HC, *Tur*.HC, *Bdi*.HC, *Osa*.HC, *Ata*_L.HC, *Ata*_J.HC, CS.HC, AK58.HC, and *Ttu*.HC) using an *E* value < 1e−10. For each gene, we selected the best-matching reference protein as a template sequence and defined the isoform sequence with maximum coverage of the template sequence as a gene representative. Genes were designated HC if they had a significant BLAST hit to reference proteins and also exceeded the coverage and identity threshold against their representative proteins in at least two references (>60% for *Osa*.HC; >65% for *Bdi*.HC; and >90% for *Hvu*.HC, *Tur*.HC, *Ata*_L.HC, *Ata*_J.HC, CS.HC, AK58.HC, and *Ttu*.HC). Second, genes with RPKM (reads per kilobase per million mapped reads) of at least 1 in the transcriptome data were also considered to be HC genes. The remaining genes were LC genes.

### Clustering and phylogenetic analysis

A total of 10 genomes from 8 species (*O. sativa*, *S. bicolor*, *Z. mays*, *S. viridis*, *B. distachyon*, *H. vulgare*, *T. aestivum* AA, *T. aestivum* BB, *T. aestivum* DD, and *H. marinum*) were selected to perform clustering and phylogenetic analysis of gene families based on a previous study (Sun et al., 2020). Estimation of the divergence time of sea barleygrass was performed using the MCMCtree program from the PAML package v4.9 (Yang, 2007). Calibration times were taken from the TimeTree database (http://www.timetree.org/). The parameters of MCMCtree were burn-in = 10 000, sample-number = 100 000, sample-frequency = 2. According to the clustering results, gene families with abnormal gene numbers in several species were filtered out, and then the expansion and contraction of gene families were analyzed with CAFE software v2.1 (https://sourceforge.net/projects/cafehahnlab/). KEGG and GO annotation of gene families was performed by aligning the genes to the KEGG database and NCBI non-redundant database using BLASTP with an *E* value of 1e−5, respectively.

### Collinearity analysis and deleterious mutant site identification

The collinearity blocks between *H. marinum* and *H. vulgare* or the three *T. aestivum* genomes/subgenomes were analyzed with MCScanX v1.1 (https://github.com/wyp1125/MCScanX), and the program jcvi (https://github.com/tanghaibao/jcvi) was used for visualization. The circos and gene-based collinearity dot plot diagrams were drawn based on the collinearity blocks and collinear gene pairs in the MCScan results. To identify deleterious mutant sites, we aligned all of the amino acid sequences in the entire genome of *H. marinum* to that of *H. vulgare* and the three subgenomes of *T. aestivum*, and we submitted the results to PROVEAN for functional variant screening (Choi and Chan, 2015). Amino acid variants with a score < −2.5 were regarded as deleterious mutant sites.

### WGD analysis and estimation of divergence time

The all-against-all BLASTP method (*E* value < 1e−5) was used to detect paralogous genes in *H. marinum*, *H. vulgare*, and the three *T. aestivum* subgenomes. Within one genome, the best (self-optimum) alignment was removed, and then the top 5 alignments of each gene were retained. Between the *H. marinum* and *H. vulgare* genomes, bidirectional pairwise alignment (A vs. B and B vs. A) was performed, and the two-way best hit was selected. MUSCLE alignments of paralogous or orthologous genes in collinear blocks were performed. Finally, the Ks value of each gene pair was calculated with KaKs_Calculator 2.0 (Wang et al., 2010), and the distribution was displayed. Complete LTR-RTs in the genomes of sea barleygrass, barley, and wheat were identified, and the insertion time was deduced (Zhang et al., 2020a, 2020b).

### Candidate gene identification and evolutionary bioinformatics

First, the protein sequences of the reported rice genes were aligned to the reference genomes of three species, and the initial candidate genes were generated based on a previous study (Sun et al., 2020). Then, PfamScan (https://www.ebi.ac.uk/seqdb/confluence/display/THD/PfamScan) was used to search the domains of the candidates and the rice genes. Identified genes with more than 50% of the domains that were also found in the rice reference genes were retained as gene set A. Then an alignment between the rice genes and the annotated genes from the reference genomes of the three species was constructed with BLASTP software, and results with an identity of at least 60% after similar filtering by PfamScan were collected into gene set B. Finally, the genomic positions of the two gene sets were compared. If they overlapped, the genes in set B were retained; if not, both were retained. The subcellular localization of SOS1 transporter candidates was predicted with Plant-mPLoc (http://www.csbio.sjtu.edu.cn/bioinf/plant-multi/), WoLF PSORT (https://wolfpsort.hgc.jp/), TargetP-2.0 (https://services.healthtech.dtu.dk/service.php?TargetP-2.0), and YLoc (https://abi-services.informatik.uni-tuebingen.de/yloc/webloc.cgi).

The Ka/Ks analysis of the candidate genes from *H. marinum*, *H. vulgare*, and *T. aestivum* was performed using the branch model of PAML v4.9 (Yang, 2007). Orthologous genes were identified based on rice gene sequences by the BLAST method. The comparative genetic similarity analysis was described previously (Zhao et al., 2019). The heatmap was generated using TBtools software v1.0 (https://github.com/CJ-Chen/TBtools/releases), and phylogenetic trees were constructed with MEGA X (https://www.megasoftware.net/) using the neighbor-joining method and polished with iTOL (https://itol.embl.de/index.shtml).

### Salt treatments and element content determination

Salt treatment of three-week-old seedlings of sea barleygrass H559 was initiated by adding NaCl to the 1/5 Hoagland solution at increments of 100 mM per day to reach final concentrations of 100, 200, 300, 400, and 500 mM. After a 1-month treatment, roots and shoots were harvested. For salt tolerance assessment of H559, Morex, and CS, salt treatment was initiated 7 days after transplanting by adding NaCl to the solution at increments of 50 and 100 mM per day to reach final concentrations of 150 and 300 mM, respectively. After 4 days of salt treatment, roots and shoots of individual plants under salt and control conditions were harvested for transcriptome sequencing. After 14 and 21 days of treatment, the plants were harvested and dried in an oven. Then the concentrations of macro-elements (Na, K, Ca, and Mg) were determined with an inductively coupled plasma optical emission spectrometer (ICP-OES) (Optima 8000, PerkinElmer, USA). Three biological replicates were sampled for high-throughput sequencing and six replicates for physiological analysis.

### Differential expression analysis of salt-tolerance-related genes

The transcriptome sequencing methods were mentioned above. The clean reads were mapped to the reference genomes of sea barleygrass (H559), barley (Morex), and wheat (CS) using HISAT2 (http://daehwankimlab.github.io/hisat2/). The read counts of each gene were calculated with HTSeq 0.9.1 (https://htseq.readthedocs.io/en/release_0.9.1/), and differential expression analysis was performed using DESeq (https://www.bioconductor.org/packages//2.10/bioc/html/DESeq.html). The Pearson’s correlation between different biological replicates was at least 0.9. Genes with FPKM ≥ 1, FDR < 0.05, and |log_2_ (treatment fpkm/control fpkm)| ≥ 2 were considered to be DEGs. Subsequently, 30 DEGs were randomly selected for qRT–PCR assays using iTaq Universal SYBR Green Supermix (Bio-Rad, USA) on a real-time PCR system (LightCycler 480 II, 96-well, Roche, Switzerland), and a correlation analysis of the RNA-seq data and qRT–PCR data was performed. Correlations between the RNA-seq and qRT–PCR data were analyzed for each plant species (supplemental Figure 17). The absolute expression of *HKT1;5* and *SOS1* was analyzed as described in a previous study (Whelan et al., 2003). All primers used in this study are listed in supplemental Table 17. GO and KEGG enrichment analyses were performed using the DAVID program (https://david.ncifcrf.gov/) and g:Profiler (https://biit.cs.ut.ee/gprofiler/).

### *Agrobacterium*-mediated transformation of immature sea barleygrass embryos

Plants were grown in a growth chamber (22°C/18°C, day/night) and in a field in Hangzhou, China. Sea barleygrass transformation was performed based on a reported method for barley and *Brachypodium* transformation, with some modifications (Bartlett et al., 2008; Vogel and Hill, 2008). In brief, immature sea barleygrass spikes were collected when the embryos were 0.5–1.0 mm in diameter. The isolated immature embryos were cultivated on improved barley callus induction medium (CI5) containing 3.65 g l^−1^ phytagel and 5 mg l^−1^ dicamba, and embryonic callus initiation was observed within 3 weeks. After an additional week, the callus was used for transformation. The standard *Agrobacterium* inoculation and co-cultivation protocol was described in the barley transformation. Here, a small drop of *Agrobacterium* suspension with OD_600_ of 0.8 was added to each callus, and they were then co-cultivated for 2 days. After co-cultivation, callus was transferred to fresh CI5 plates containing 25 mg l^−1^ hygromycin and 200 mg l^−1^ timentin. After 4 weeks of selection culture, callus was transferred to a transition medium (T5) containing 2.7 g l^−1^ Murashige and Skoog modified plant salt base (without NH_4_NO_3_) (Duchefa M0238), 20 g l^−1^ maltose, 825 mg l^−1^ NH_4_NO_3_, 750 mg l^−1^ glutamine, 690 mg l^−1^ proline, 500 mg l^−1^ casein hydrolysate, 100 mg l^−1^ myo-inositol, 0.4 mg l^−1^ thiamine HCl, 0.15 mg l^−1^ 2,4-dichlorophenoxy acetic acid (2,4-D), 5 mg l^−1^ kinetin (KT), 1.25 mg l^−1^ CuSO_4_·5H_2_O, and 3.4 g l^−1^ phytagel with 20 mg l^−1^ hygromycin and 180 mg l^−1^ timentin in low light (75 μmol m^−2^ s^−1^). After a further 2 weeks, embryo-derived callus was transferred to regeneration medium, which was the same as the T5 medium but without additional copper. Regenerated plants with shoots of 2–3 cm in length were transferred to test tubes containing CI medium without dicamba or any other growth regulators but still containing 5 mg l^−1^ hygromycin and 160 mg l^−1^ timentin. Transformed plants developed a strong root system in the hygromycin-containing medium in 1–2 weeks and were then transferred to soil.

### CRISPR/Cas9-mediated sea barleygrass genome editing system

The gRNA target site for the *HmSOS1* gene was cloned into the pUB-Cas9-TaU6-sgRNA vector in which *SpCas9* was driven by the maize *Ubi* promoter and the sgRNA expression cassette was driven by the *TaU6* promoter (Lawrenson and Harwood, 2019). A schematic diagram of the vector is presented in Figure 6B. Transformation of sea barleygrass cells with *Agrobacterium* AGL1 containing the target recombinant plasmid yielded regenerated plants. The specific primers Cas9-F/R were then used to detect the presence of the T-DNA insertion event in the genome. The single-allelic and biallelic mutants were subsequently verified by PCR/RE assays. The sgRNA target genomic DNA region containing the *Bse*RⅠ recognition site was amplified by a PCR assay in all gene edited plants. After digestion with restriction enzymes, the mutations induced by the genome editing system formed uncleaved bands in the agarose gel because of the loss of restriction sites. The biallelic, heterozygous, and homozygous mutations were further confirmed by Sanger sequencing and analyzed using the program DSDecode (Liu et al., 2015).

### Data and materials availability

All raw transcriptomic data generated from the three Triticeae species and genome sequencing data for *H. marinum* accession H559 have been deposited into the Sequence Read Archive (SRA) database at NCBI under BioProject accessions PRJNA639318 and PRJNA597957, respectively. The genome assembly and annotation results have been submitted to the Genome WareHouse (GWH) database at the China National Genomics Data Center with BioProject accession number PRJCA009391. All other data are available in the main text or the supplemental information.

## Funding

This research was supported by The National Key Research and Development Program of China (2018YFD1000704), the 10.13039/501100001809National Natural Science Foundation of China (32071934), the key research project of Zhejiang (2020C02002, 2021C02064-3), the China Agriculture Research System of MOF and MARA, and the 10.13039/501100018522Jiangsu Collaborative Innovation Center for Modern Crop Production.

## Supplemental information

Document S1. Supplemental Figures 1–25 and supplemental Tables 1–17

Document S2. Supplemental Datasets 1–5

Document S3. Article plus supplemental information
