# Supplementary material for: The genome and gene editing system of sea barleygrass provide a novel platform for cereal domestication and stress tolerance studies
Source: Plant Commun. 2022 May 5;3(5):100333. doi: 10.1016/j.xplc.2022.100333 (PMC9482977; doi:10.1016/j.xplc.2022.100333)
Supplement: Document S1. Supplemental Figures 1–25 and supplemental Tables 1–17 [file mmc1.pdf]

Plant Communications, Volume *3*

Supplemental information

The genome and gene editing system of sea barleygrass provide a novel platform for cereal domestication and stress tolerance studies

Liuhui Kuang, Qiufang Shen, Liyang Chen, Lingzhen Ye, Tao Yan, Zhong-Hua Chen, Robbie Waugh, Qi Li, Lu Huang, Shengguan Cai, Liangbo Fu, Pengwei Xing, Kai Wang, Jiari Shao, Feibo Wu, Lixi Jiang, Dezhi Wu, and Guoping Zhang

Supplemental information

The genome and gene editing system of sea barleygrass provide a novel platform for cereal domestication and stress tolerance studies

Liuhui Kuang, Qiufang Shen, Liyang Chen, Lingzhen Ye, Tao Yan, Zhong-Hua Chen, Robbie Waugh, Qi Li, Lu Huang, Shengguan Cai, Liangbo Fu, Pengwei Xing, Kai Wang, Jiari Shao, Feibo Wu, Lixi Jiang, Dezhi Wu, Guoping Zhang

This PDF file includes:

Supplemental Figures 1-25
Supplemental Tables 1-17

Other supplemental information for this manuscript includes the following:

Supplemental Datasets 1-5

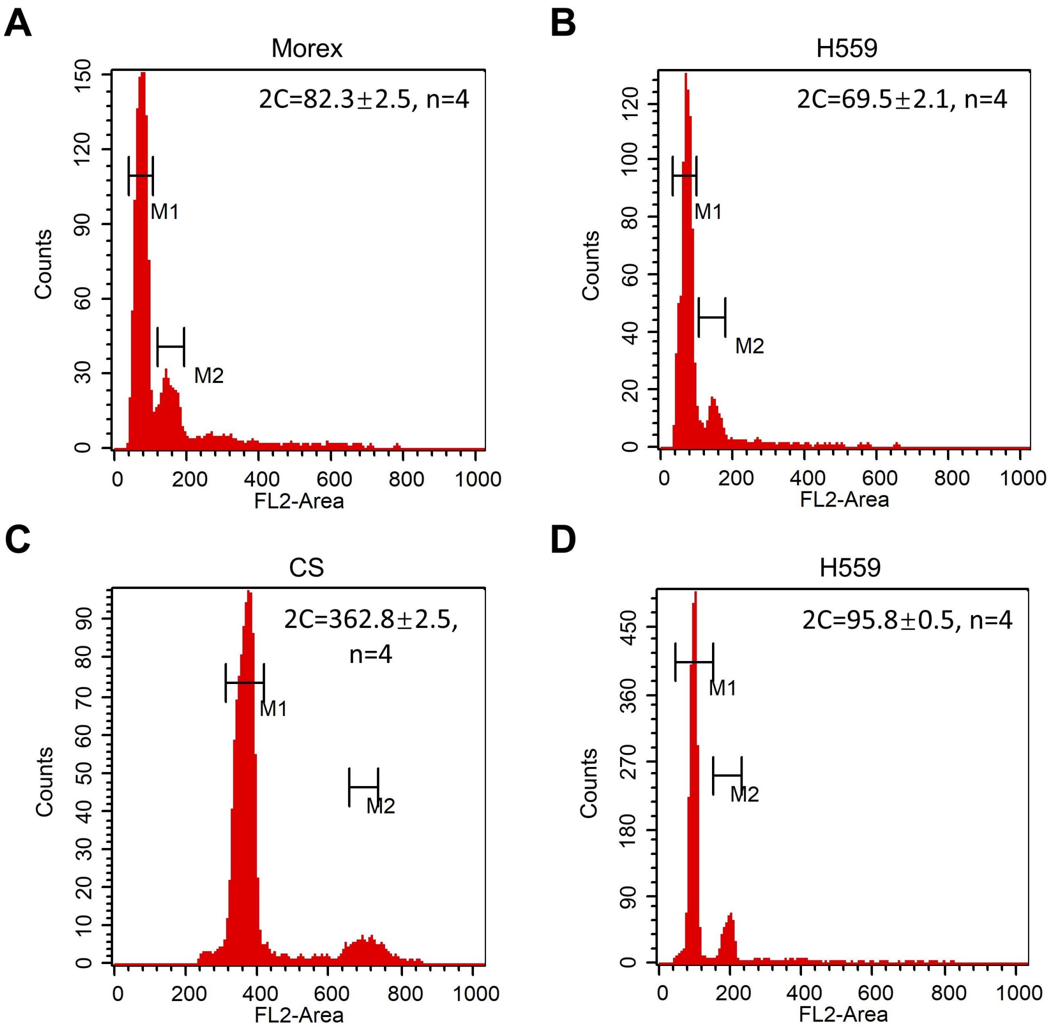

Supplemental Figure 1. The H559 genome size estimation using FCM (flow cytometry).

(A-B) The FCM analysis of sea barleygrass accession H559 when barley (cv. Morex; Genome size: ~5.1 Gb) was used as an internal control. **(C-D)** The FCM analysis of sea barleygrass accession H559 with wheat (cv. Chinese Spring, CS; Genome size: 15.4-15.8 Gb) as an internal control. Four biological replicates were set and 2C values were shown as mean ± SD (n=4).

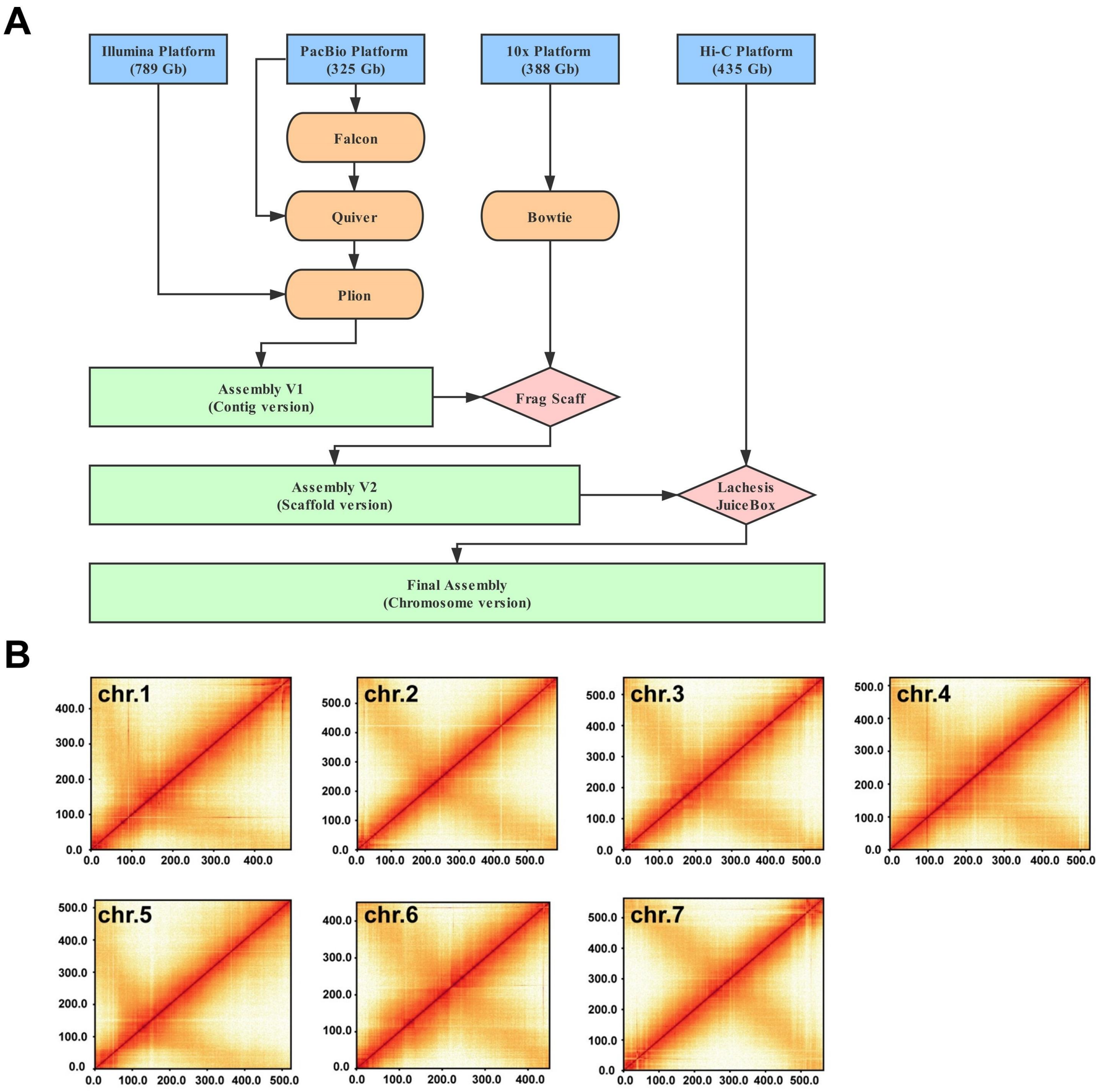

Supplemental Figure 2. Flow diagram of the genome sequencing methods (A) and the heatmap of seven chromosomes (B) by Hi-C in the sea barleygrass genome.

LACHESIS software was used to perform Hi-C-assisted assembly of the genome, and finally seven long-clustering sequences were obtained, corresponding to seven chromosomes.

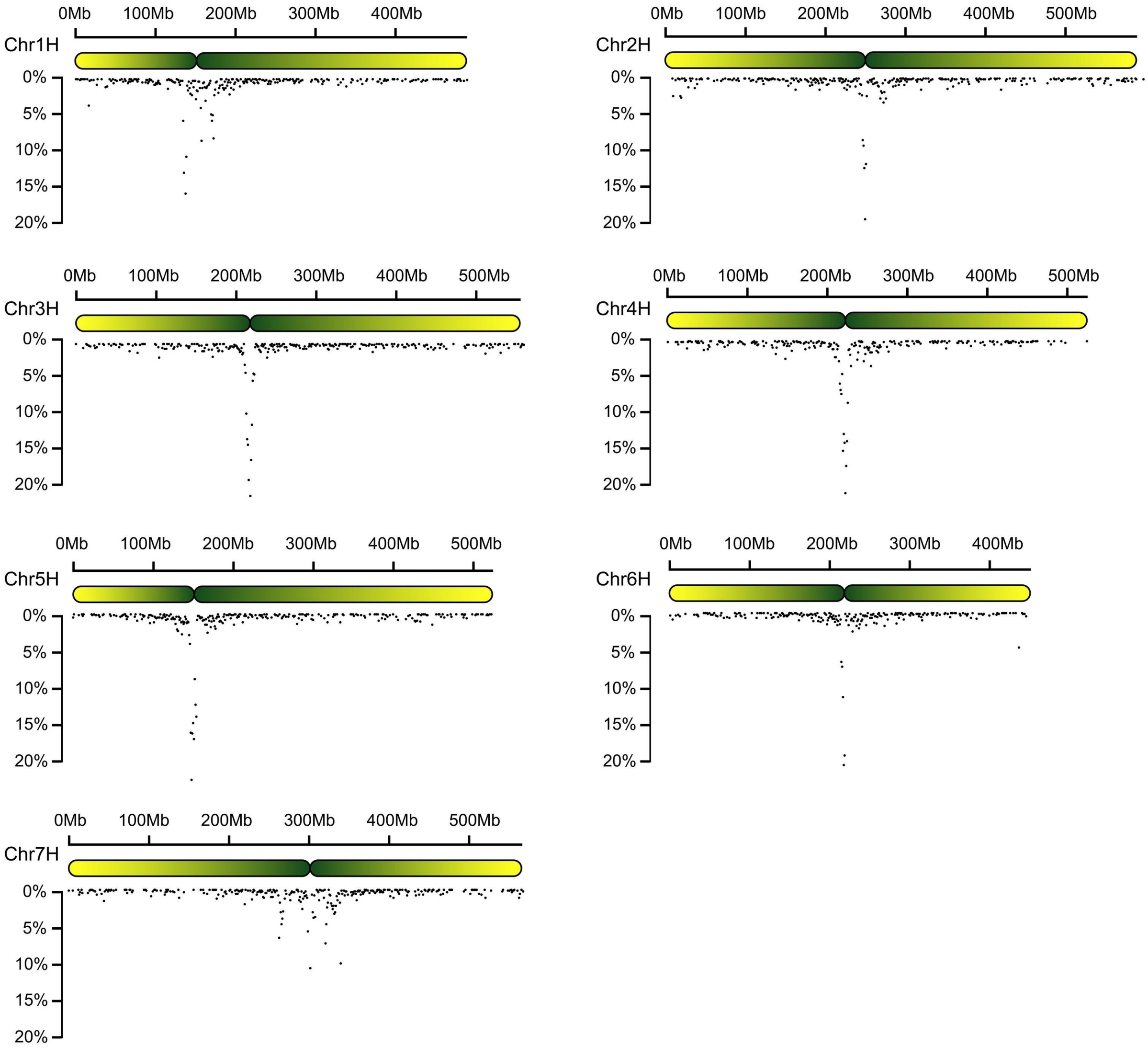

Supplemental Figure 3. The density distribution of *Cereba* and *Quinta* cross each chromosome of *H. marinum*.

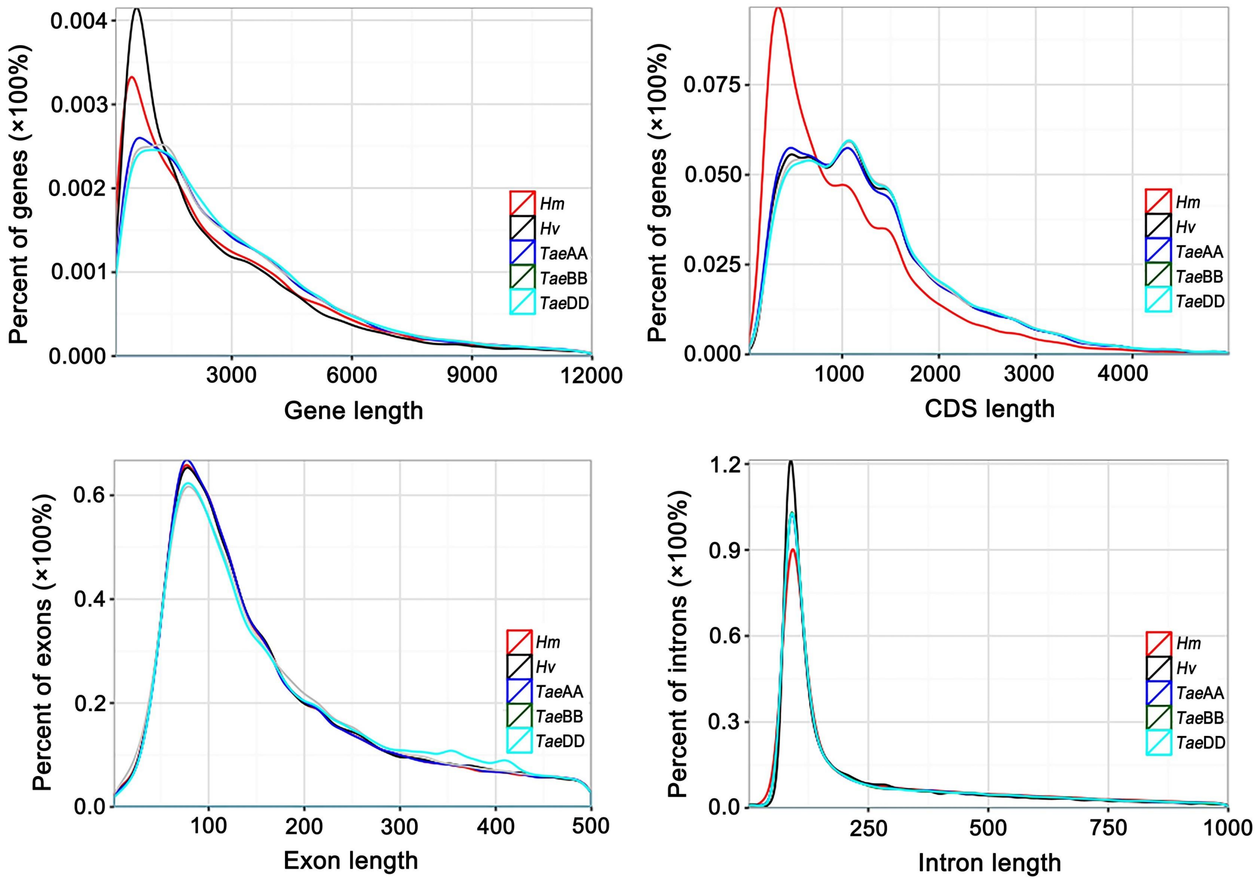

Supplemental Figure 4. Comparison of gene structural features of the *H. marinum* genome to the *H. vulgare* and *T. aestivum (*AA, BB and DD subgenomes) genomes.

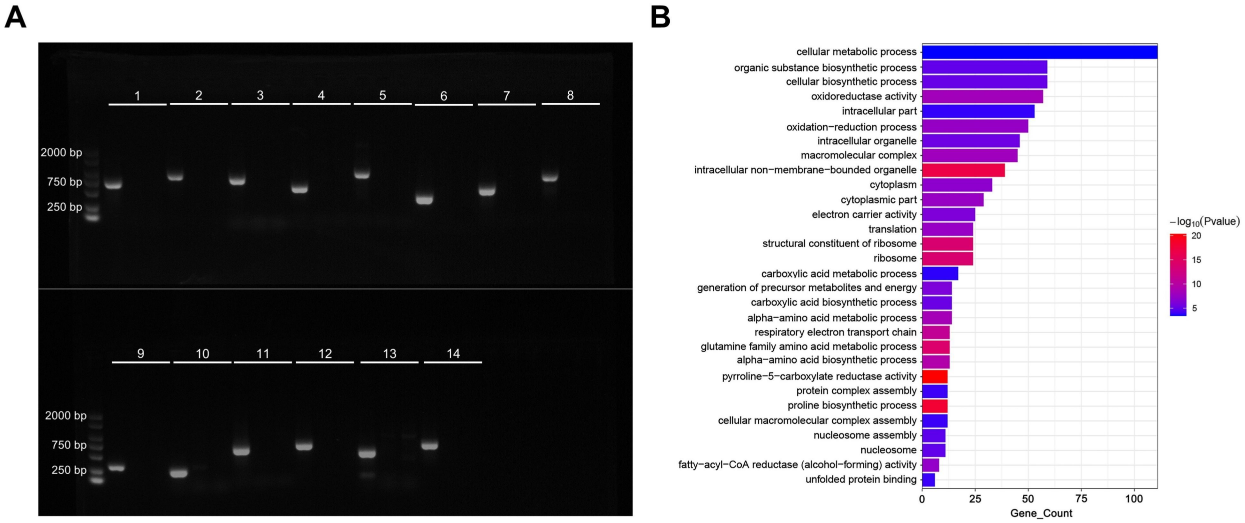

Supplemental Figure 5. PCR amplification (A) and GO enrichment (B) of putatively unique genes in the sea barleygrass genome.

Compared with the genomes of *H. vulgare* and three subgenomes of *T. aestivum*, there were 941 unique gene families in the sea barley genome. Among them, 14 unique genes from 7 chromosomes were randomly selected for PCR amplification using gDNA of sea barleygrass (accession H559), barley (cv. Morex) and wheat (cv. CS). The primers used for PCR were listed in Supplemental Table 17.

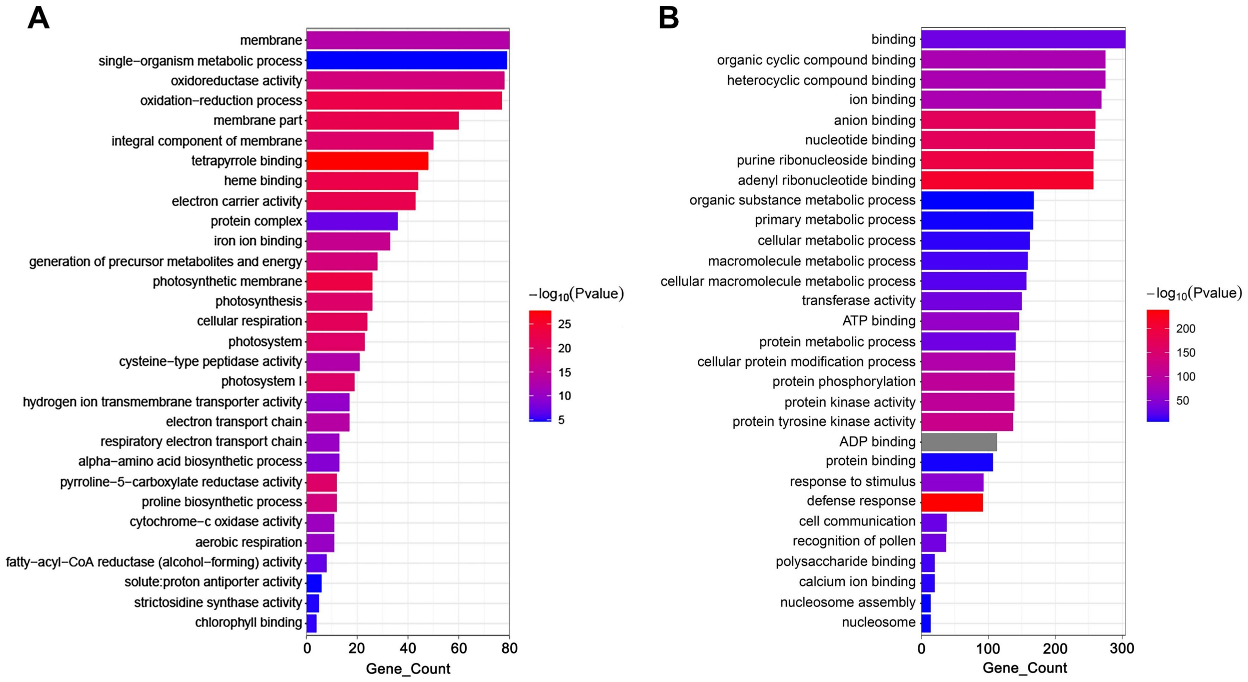

Supplemental Figure 6. GO enrichment of expanded (A) and contracted (B) gene families in the sea barleygrass genome.

Compared with the common ancestor of barley, 254 gene families in the sea barley genome had expanded, and 111 gene families had contracted, which was analyzed by CAFÉ (http://sourceforge.net/projects/cafehahnlab/).

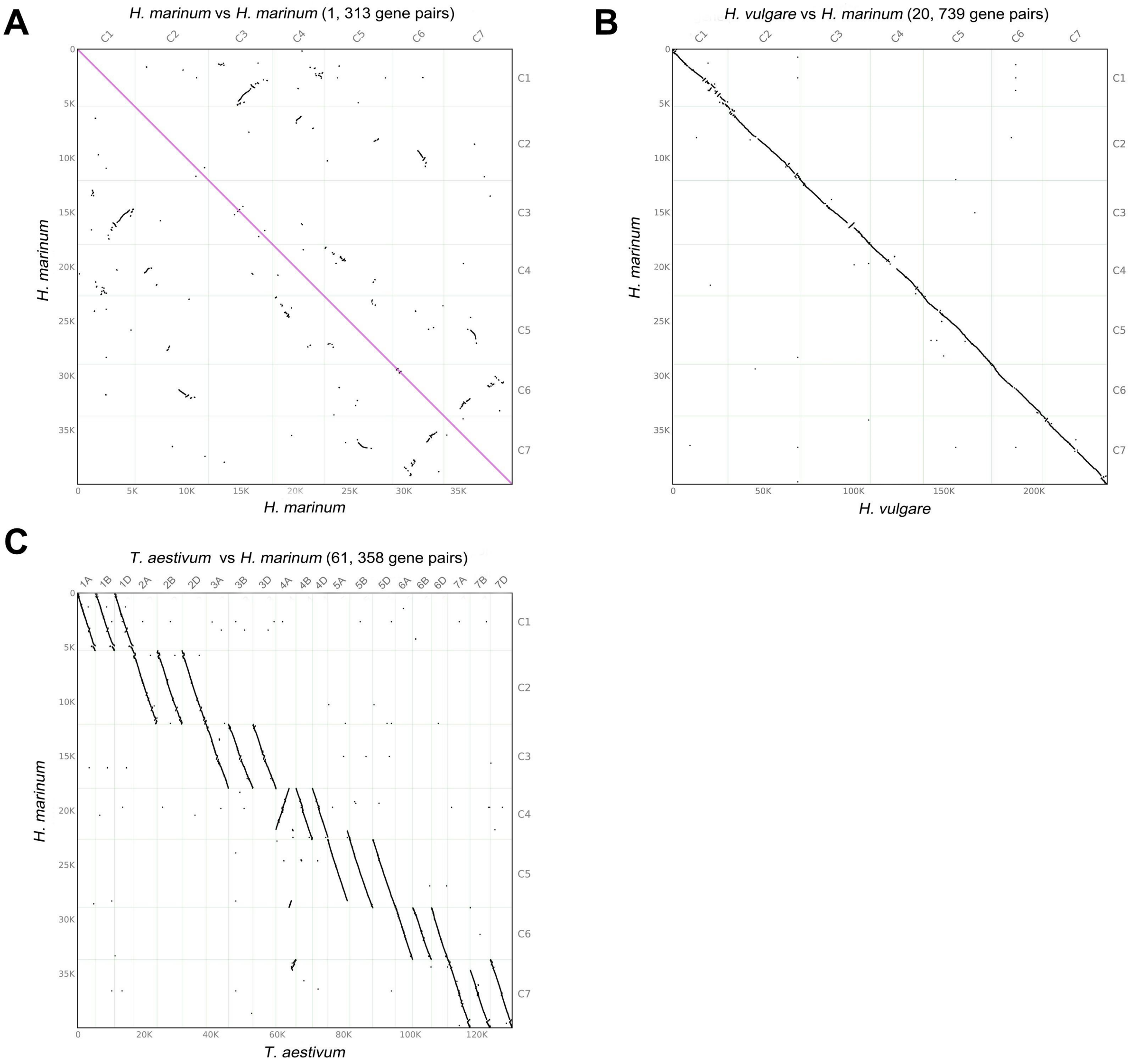

Supplemental Figure 7. The gene-based collinearity dot plot comparison among *H. marinum*, *H. vulgare* and *T. aestivum* genomes.

(A) The inter-genomic comparison within *H. marinum* genome. **(B)** The inter-genomic comparison between *H. vulgare* and *H. marinum.* **(C)** The inter-genomic comparison between *T. aestivum* and *H. marinum.*

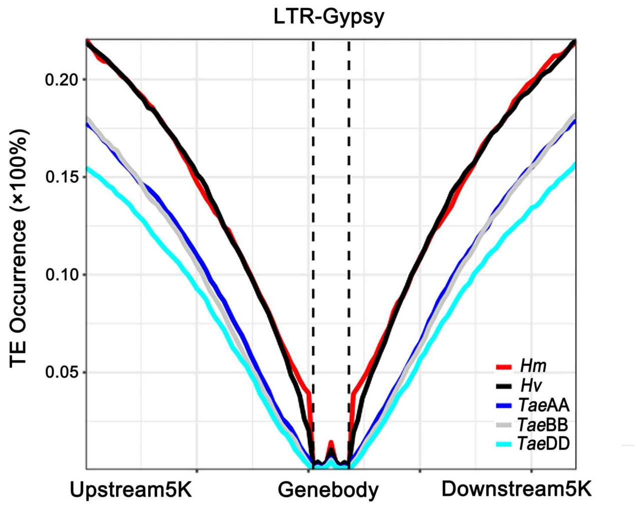

Supplemental Figure 8. The occurrence of LTR-Gypsy and LTR-Copia in the upstream and downstream (5 kb) and gene body regions in the genomes of sea barleygrass, barley and wheat.

Different color lines indicate different genomes.

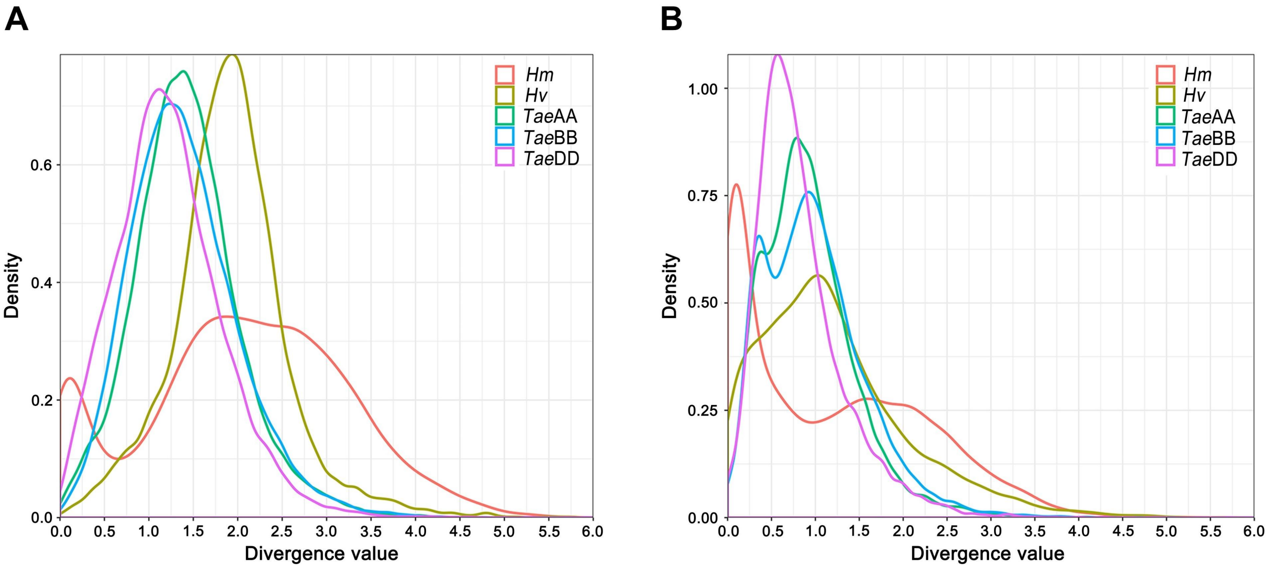

Supplemental Figure 9. The estimated insertion time of LTR-Copia (A) and LTR-Gypsy (B) in the genomes of sea barleygrass, barley and wheat.

The estimated insertion time (Mya) was calculated by T = K/2r (r = 1.3×10^{-8}) and corrected by JC69 model. Difference color lines indicate the different genomes.

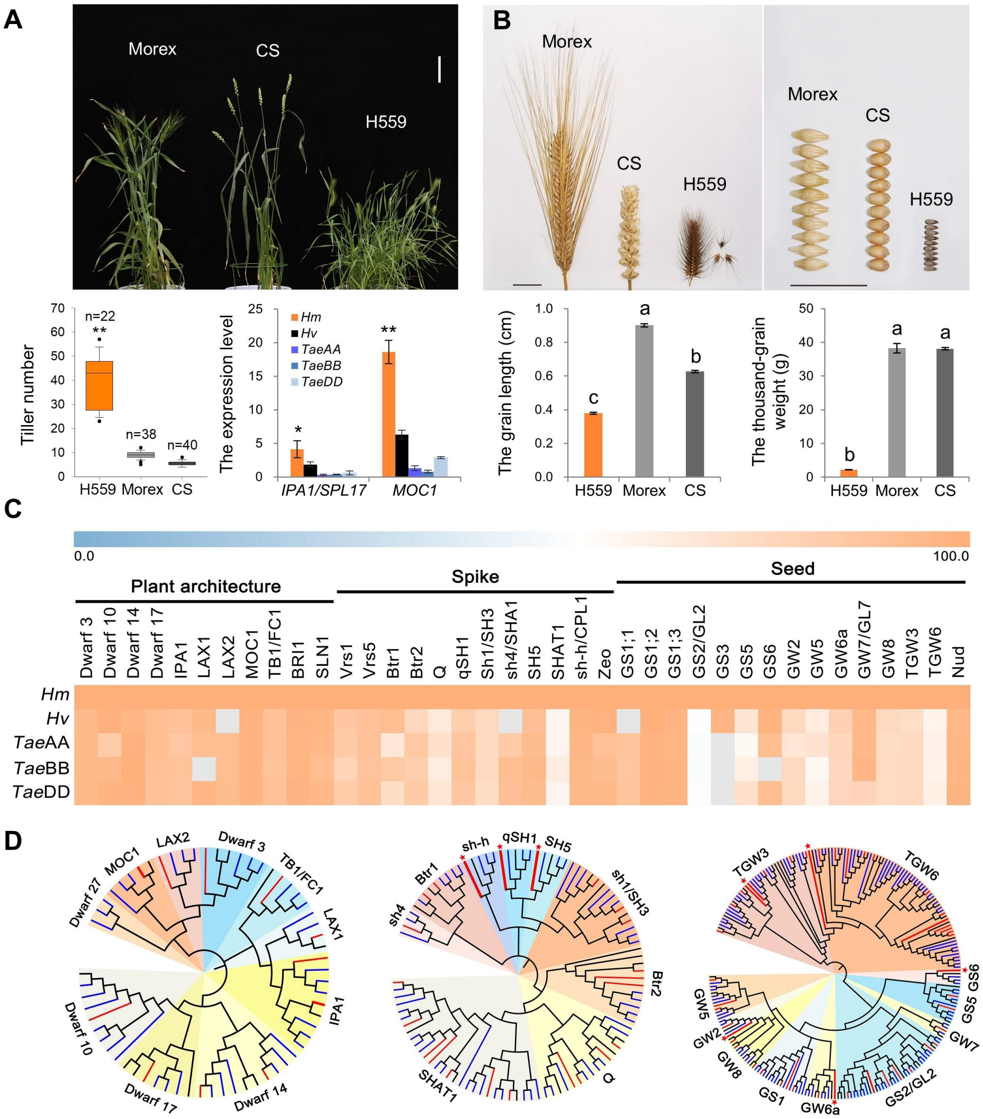

Supplemental Figure 10. Morphological divergence in sea barleygrass, barley and wheat.

(A) The picture of plant morphology, fertile tiller numbers and the expression levels of *IPA1/SPL17* and *MOC1* in barley cultivar Morex, wheat cultivar CS and sea barleygrass accession H559. Bar=10 cm. The expression level of *IPA1/SPL17* and *MOC1* was analyzed using shoot tissues from one-month-old seedlings of H559, Morex and CS. **(B)** The spike and grain morphology of Morex, CS and H559. Bar=2 cm. Grain length and grain weight of three species were compared. Data are shown as mean ± SD (10 grains as one repetition, n=10). Different letters indicate a significant difference ($P < 0.01$) using Tukey's test after a one-way ANOVA. **(C)** Heat map for amino acids similarity and the evolution of proteins involved in plant architecture, spike and seed morphology. Geneious software was used to estimate the similarity of proteins using *H. marinum* sequences as the query with E-value < 10^{-5}. Colored squares: 0 (blue), 100% (orange), no proteins satisfied the selection criterion (gray). **(D)** Phylogenetic trees of proteins involved in tillering, grain size and grain shattering. The red color indicates sea barleygrass, black indicates barley and blue indicates wheat.

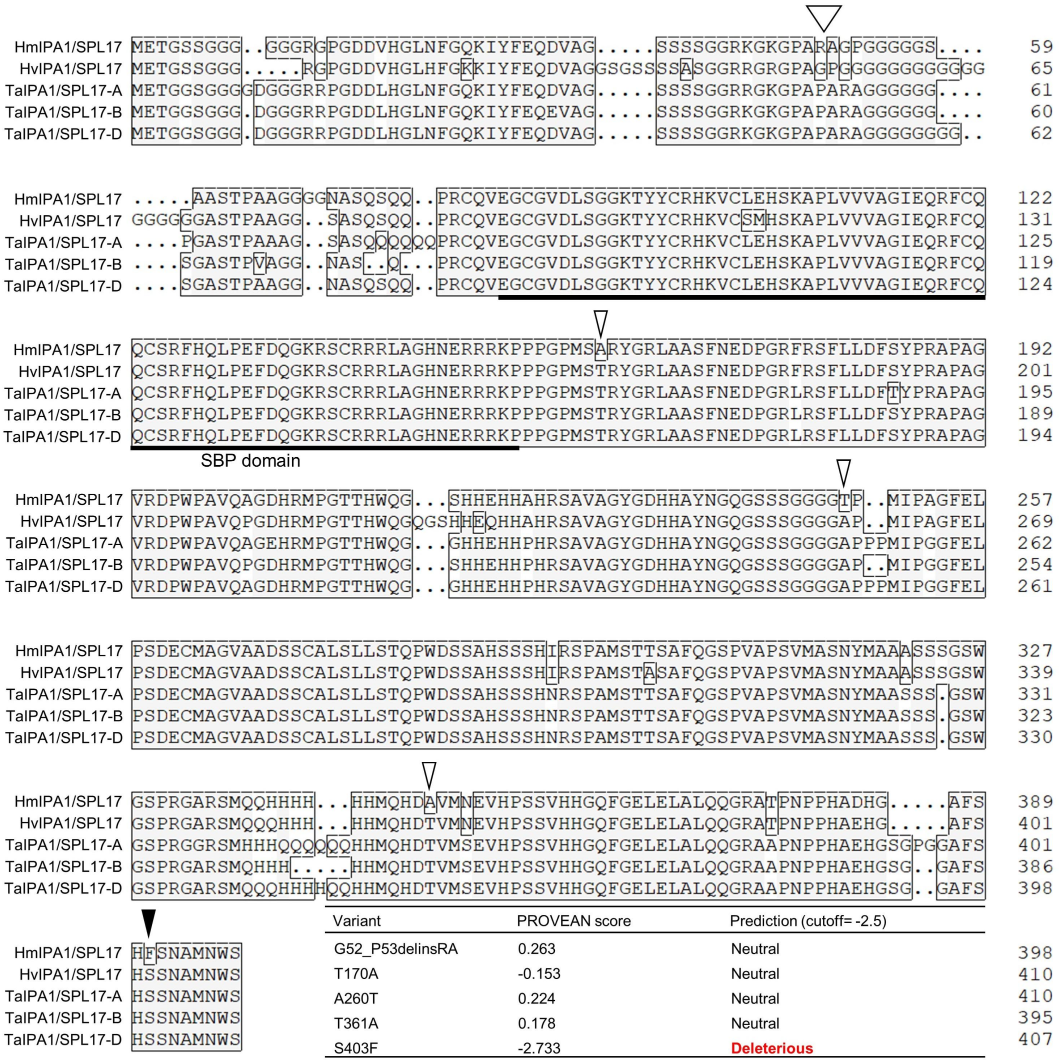

Supplemental Figure 11. The alignment of IPA1/SPL17 amino acid sequences in sea barleygrass (H559), barley (Morex) and wheat (CS).

The conserved SBP domain in IPA1 is searched in the domain database from NCBI and the neutral/deleterious variants in the protein sequence are highlighted by PROVEAN.

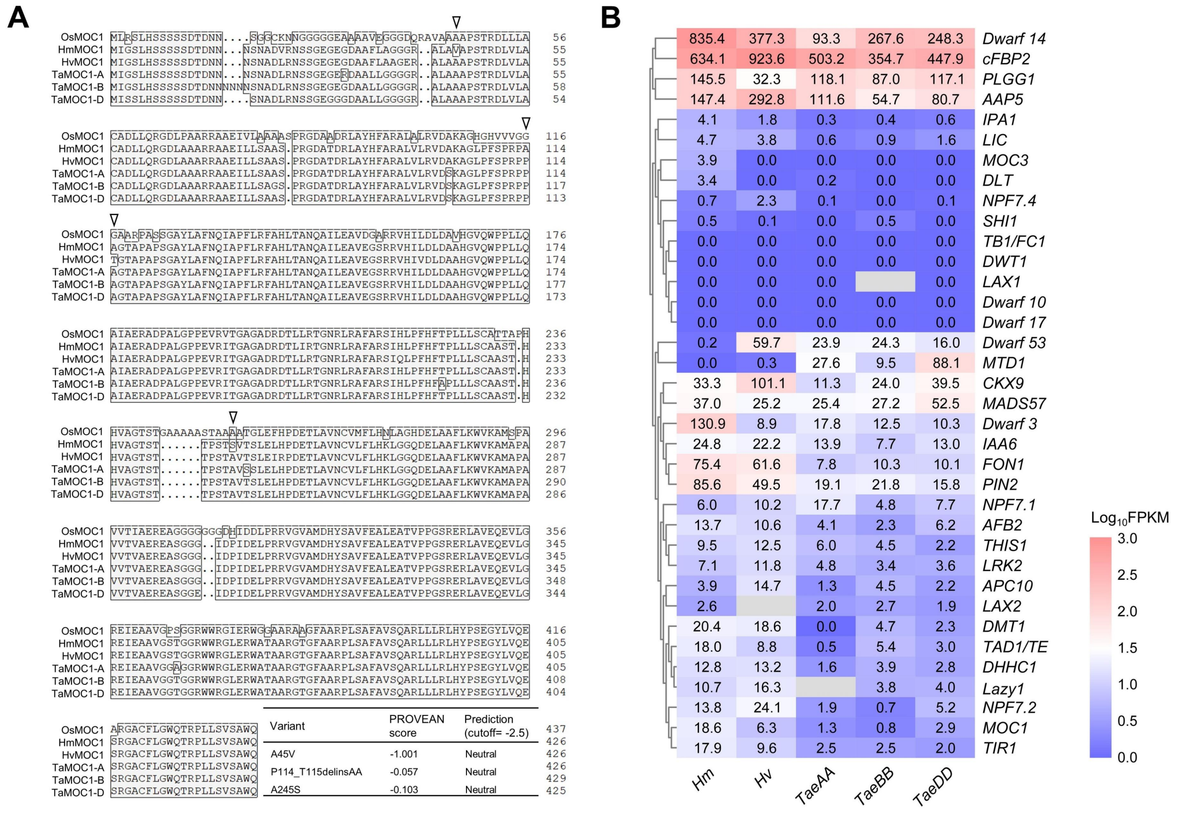

Supplemental Figure 12. The analysis of the tillering-related genes in sea barleygrass, barley and wheat.

(A) The alignment of MOC1 amino acid sequences in rice, sea barleygrass, barley and wheat. The neutral/deleterious variants in protein sequence are highlighted by PROVEAN. **(B)** The expression level of the tillering-related genes in one-month-old seedlings of sea barleygrass (H559), barley (Morex) and wheat (CS). The values are the means (n=3) of FPKM for homologous genes of three species.

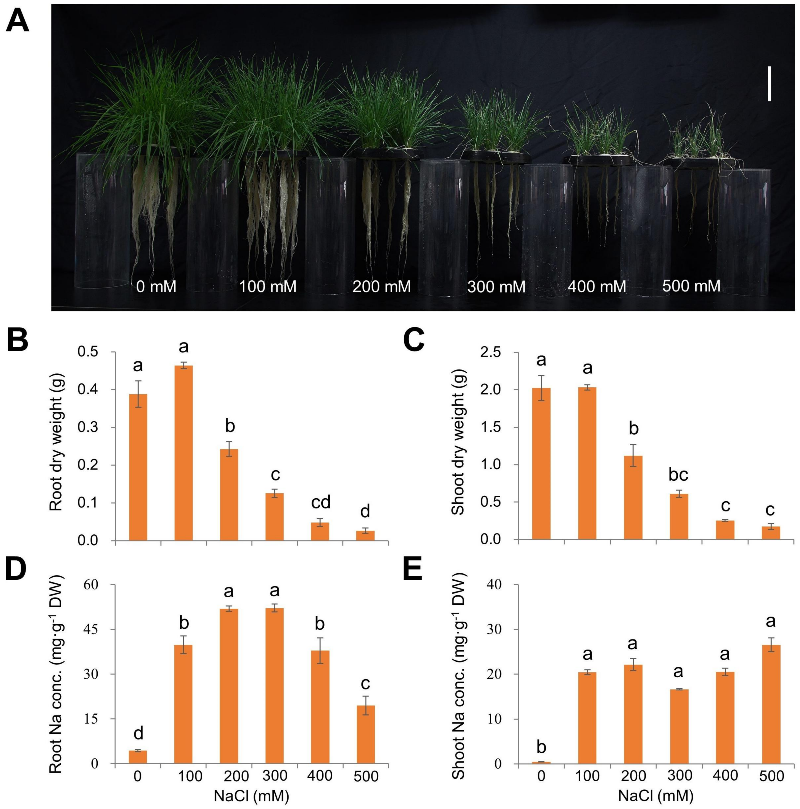

Supplemental Figure 13. The salt tolerance of sea barleygrass accession H559.

Plant growth **(A)** of sea barleygrass accession H559 after 30 d of 0, 100, 200, 300, 400 and 500 mM NaCl treatments. Bar=10 cm. Dry weight **(B and C)** and Na^+ concentration **(D and E)** in roots **(B and D)** and shoots **(C and E)**. Data are shown as mean ± SD (n=6). Different small letters indicate a significant difference ($P < 0.05$) using Tukey's test after a one-way ANOVA.

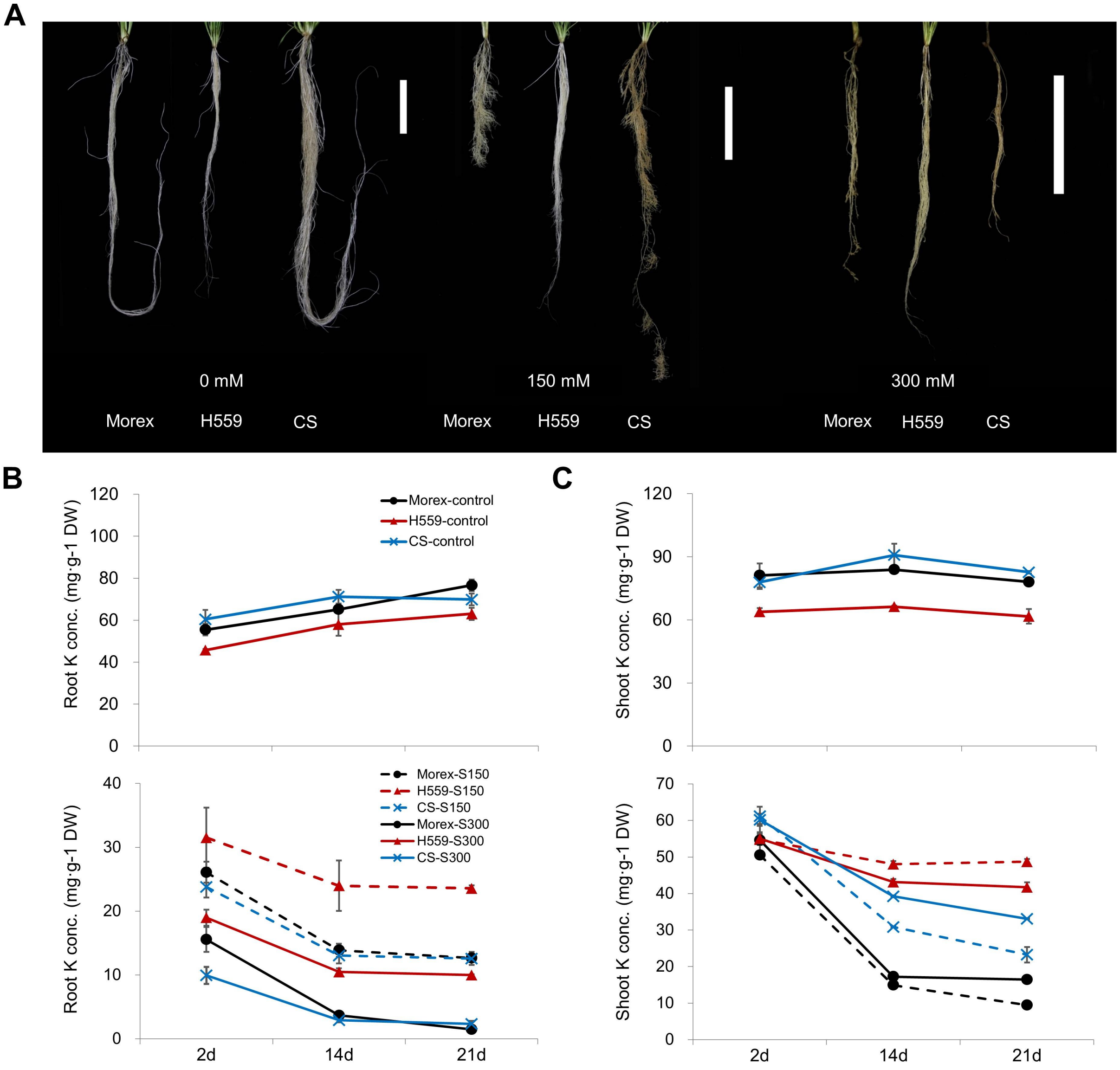

Supplemental Figure 14. Root growth (A) and K^+ concentration in the roots (B) and shoots (C) of barley (Morex), sea barleygrass (H559) and wheat (CS) under control and salt conditions.

Each genotype was grown in hydroponics. Salt stress was initiated with 150 and 300 mM NaCl to two-week-old seedlings for 2, 14 and 21 d. CS: Chinese Spring. Bar=10 cm. Data are shown as mean ± SD (n=6).

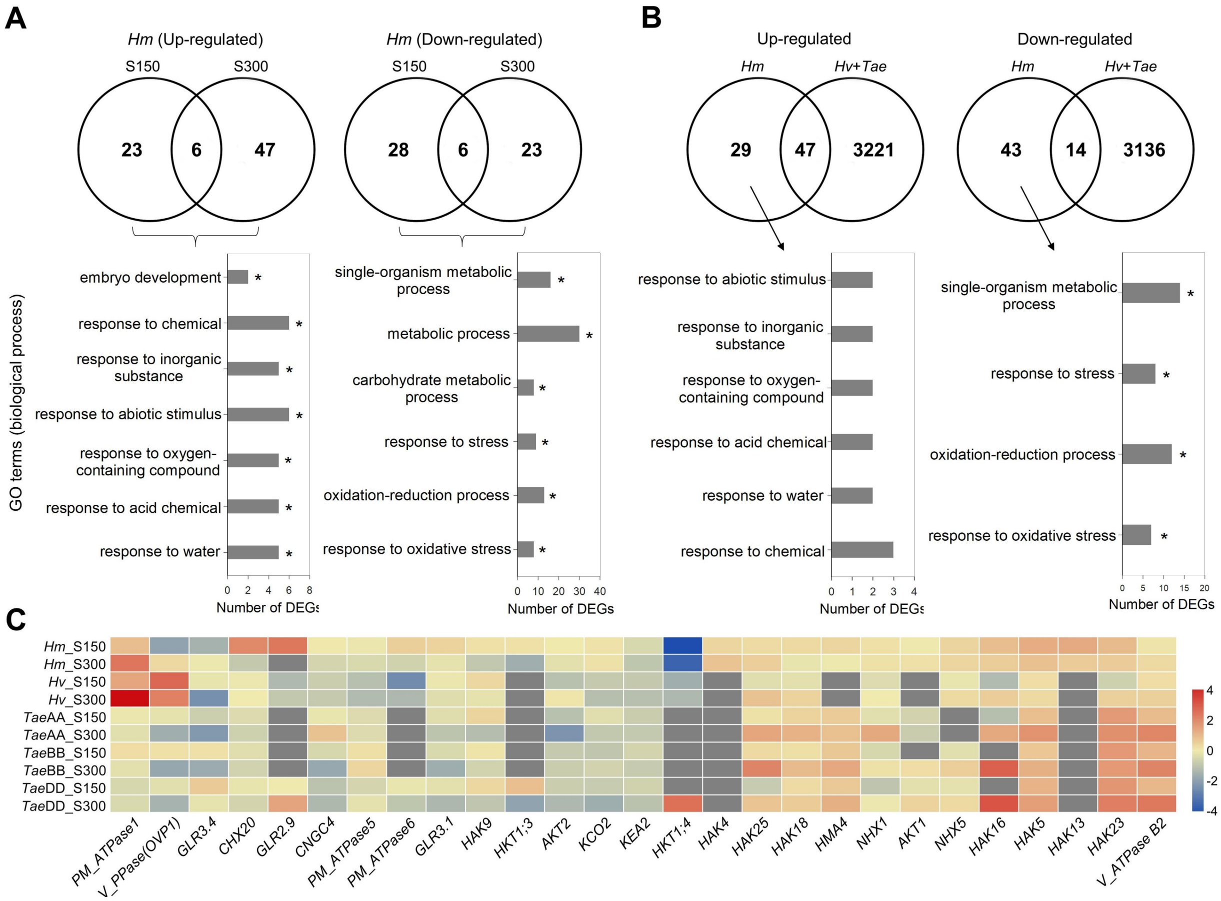

Supplemental Figure 15. Transcriptomic analysis of the shoots in H559, Morex and CS in response to salt treatments.

(A) Differentially expressed genes (DEGs) and GO enrichments (biological process) in the shoots of H559 after 4 d of 150 and 300 mM salt treatments. **(B)** DEGs and GO enrichments (biological process) in the shoots between H559 and Morex/CS. **(C)** Heatmap of the fold changes of DEGs related to ion homeostasis in the shoots of three species after 4 d of 150 (S150) and 300 (S300) mM salt treatments.

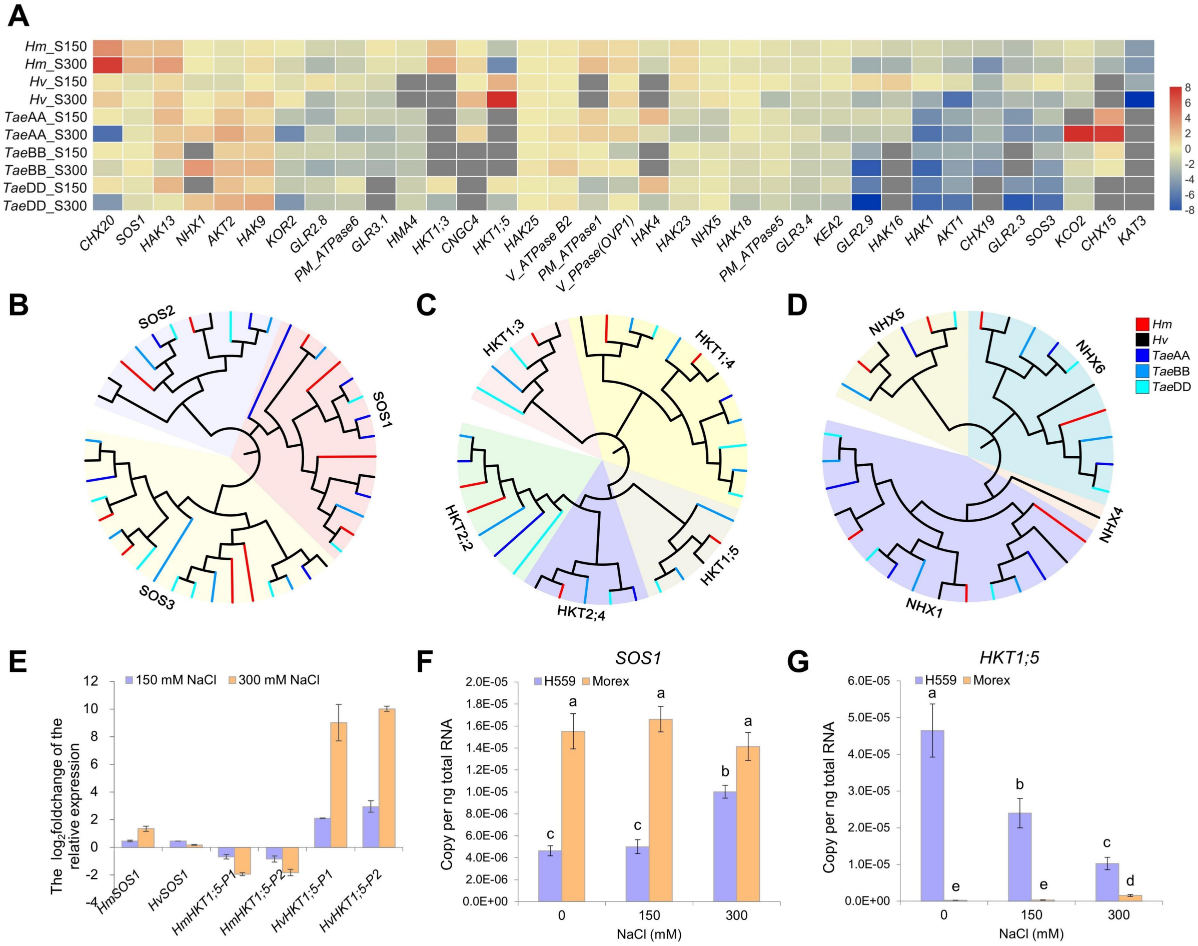

Supplemental Figure 16. Transcriptomic analysis of the roots in H559, Morex and CS in response to salt treatments.

(A) Heatmap of the fold changes of DEGs related to ion homeostasis in the roots of three species after 4 d of 150 mM (S150) and 300 mM (S300) salt treatments. The values are shown as $\log_2$ (treatment_fpkm/ control_fpkm). **(B-D)** Phylogenetic trees for genes encoding SOS pathway members **(B)**, HKT transporters **(C)** and NHX transporters **(D)** in the genomes of sea barleygrass, barley and wheat. **(E)** The relative expression analysis for *SOS1* and *HKT1;5* in sea barleygrass accession H559 and barley cv. Morex roots under salt stress. The absolute expression analysis for *SOS1* **(F)** and *HKT1;5* **(G)** in H559 and Morex roots under salt stress. 'P1' and 'P2' indicate Primer pair 1 and 2, respectively. Data are shown as mean ± SD (n=4). Different small letters indicate a significant difference ($P < 0.05$) using Tukey's test after a one-way ANOVA.

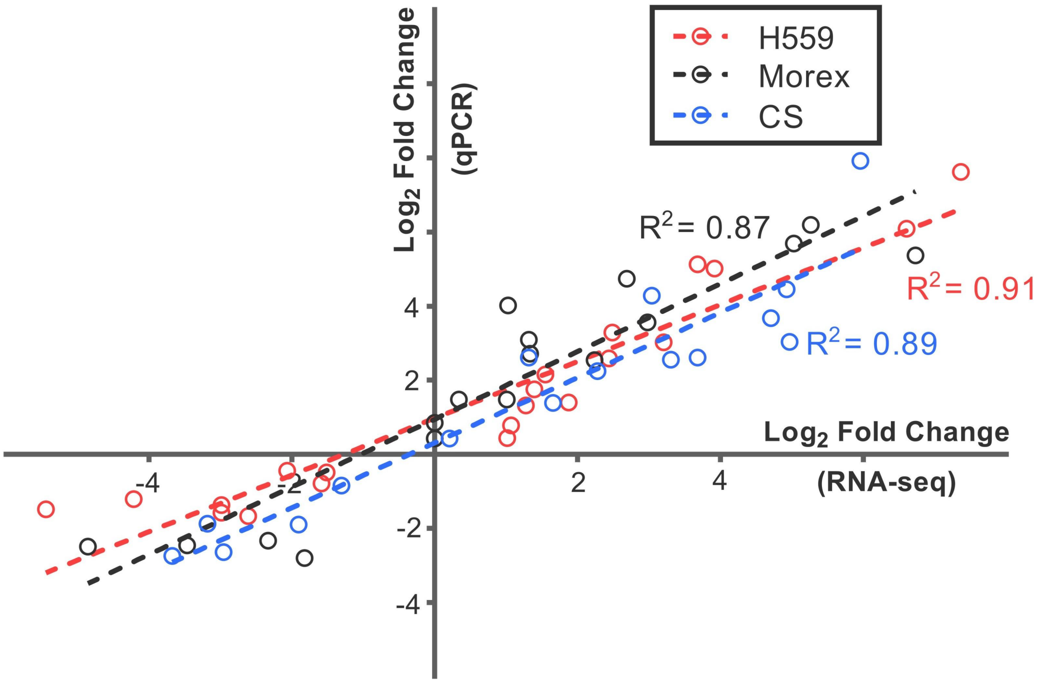

Supplemental Figure 17. The concordance of RNA-seq and RT-qPCR data in H559, Morex and CS.

Correlation of RNA-seq (x-axis) and RT-qPCR data (y-axis) using the $\log_2$ fold change measure of the genes differentially expressed *P*-value across the two gene-expression platforms under correlation analysis.

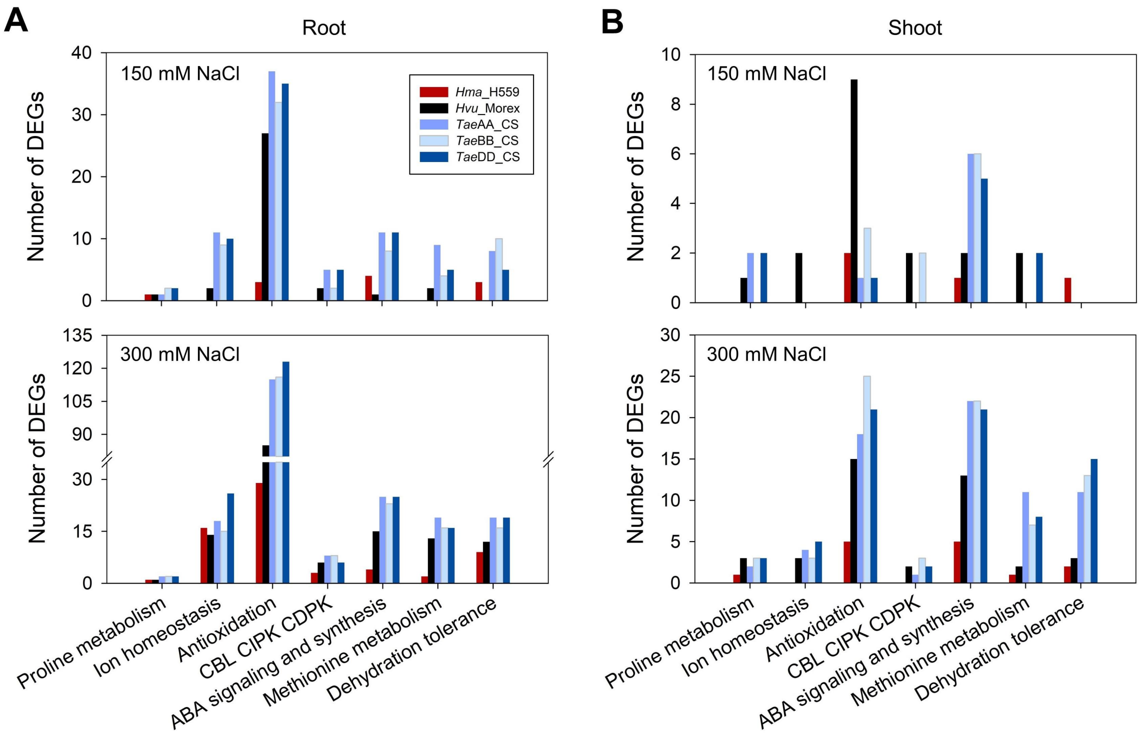

Supplemental Figure 18. The number of salt-tolerant DEGs in the roots (A) and shoots (B) of H559, Morex and CS after 4 d of 150 and 300 mM salt treatments.

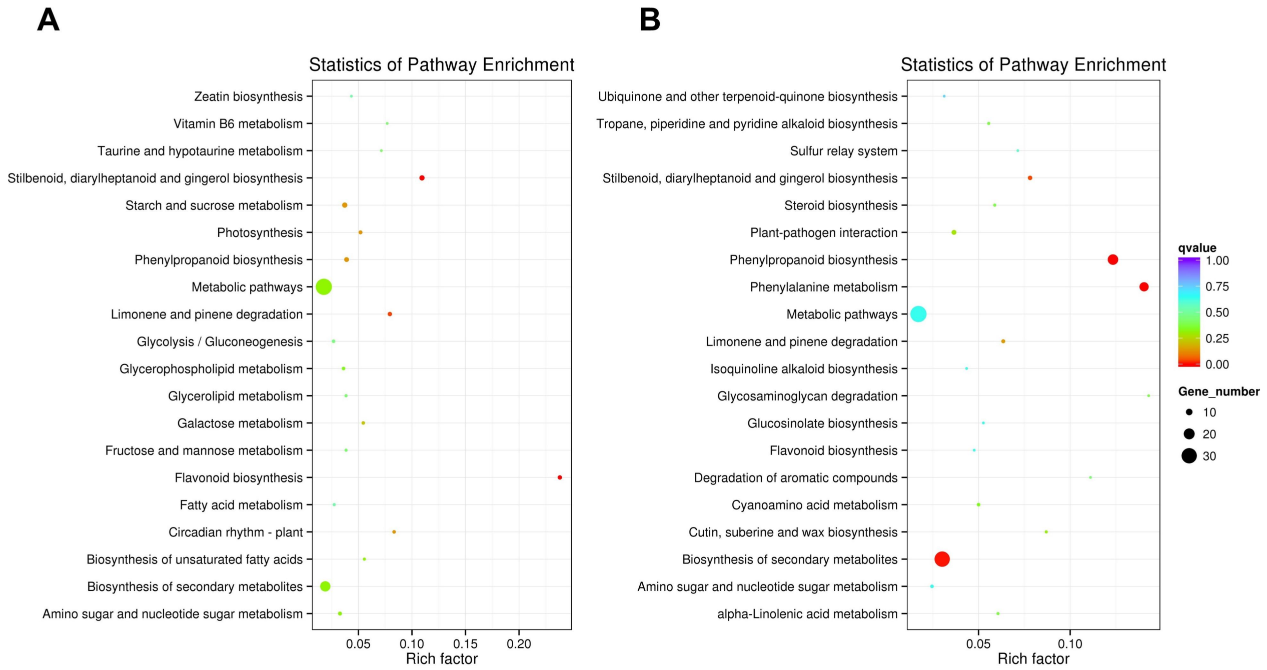

Supplemental Figure 19. KEGG enrichment of 450 specifically up-regulated (A) and 597 specifically down-regulated (B) DEGs in H559 in the roots.

Phenylpropanoid biosynthesis and phenylalanine metabolism were dramatically depressed and flavonoid biosynthesis was significantly enhanced.

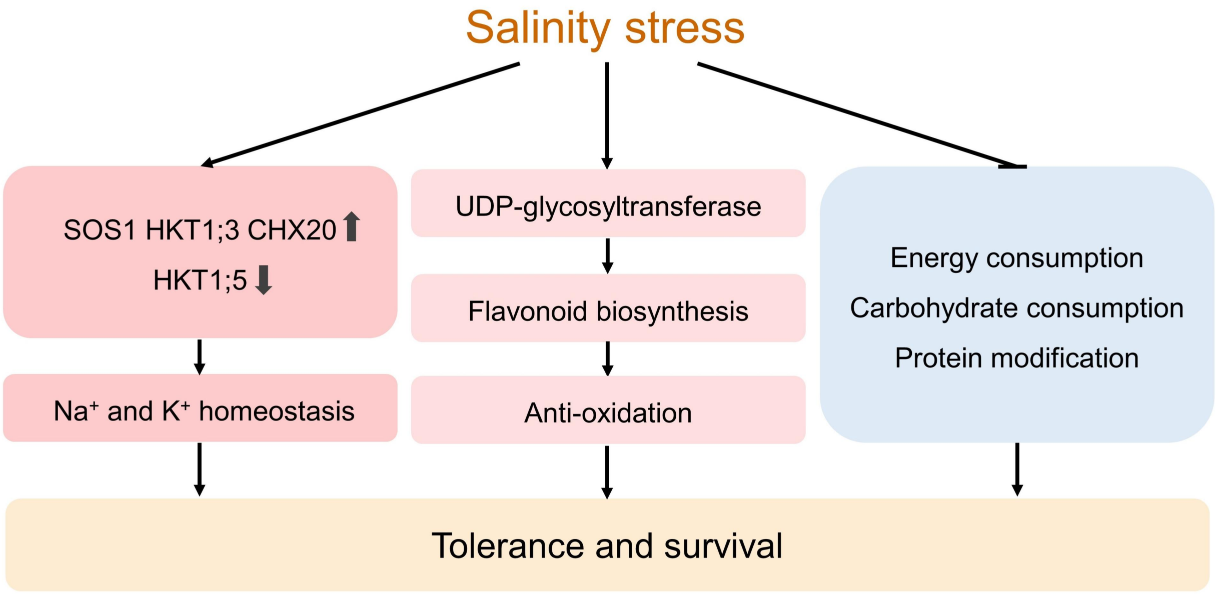

Supplemental Figure 20. The putative salt-tolerant strategies in sea barleygrass.

Under salinity stress, sea barleygrass maintained the superior Na^+ /K^+ homeostasis in roots by regulating the ion transporter genes mainly including *SOS1*, *HKT1;3*, *HKT1;5* and *CHX20*. Meanwhile, UDP-glycosyltransferase activity was enhanced, which might be responsible for the redirection of metabolic flux to flavonoid biosynthesis and the accumulation of flavonoid glycosides. In addition, suppressed protein modification and decreased energy and carbohydrate consumption also played a role in plant survival under salinity condition.

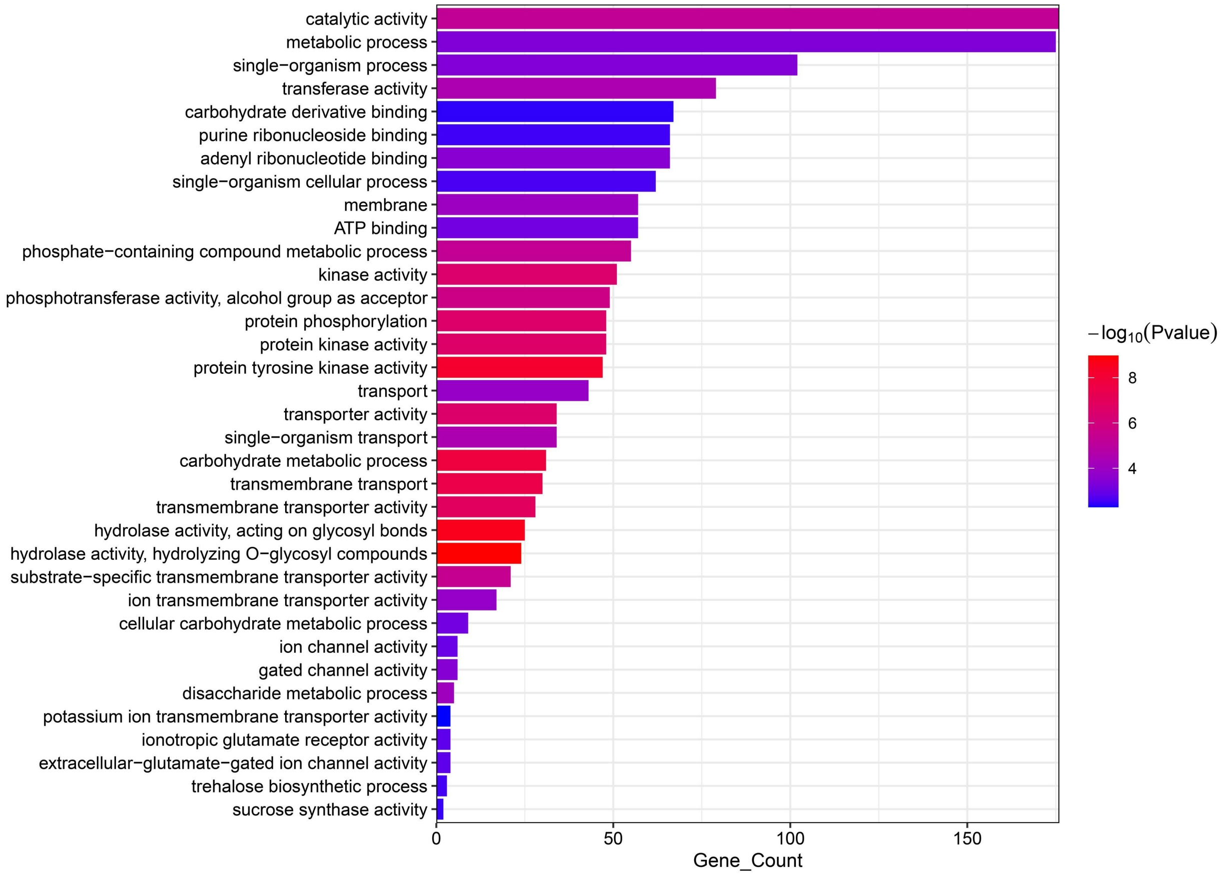

Supplemental Figure 21. GO enrichment analysis of 406 DEGs with deleterious amino acid variants in H559.

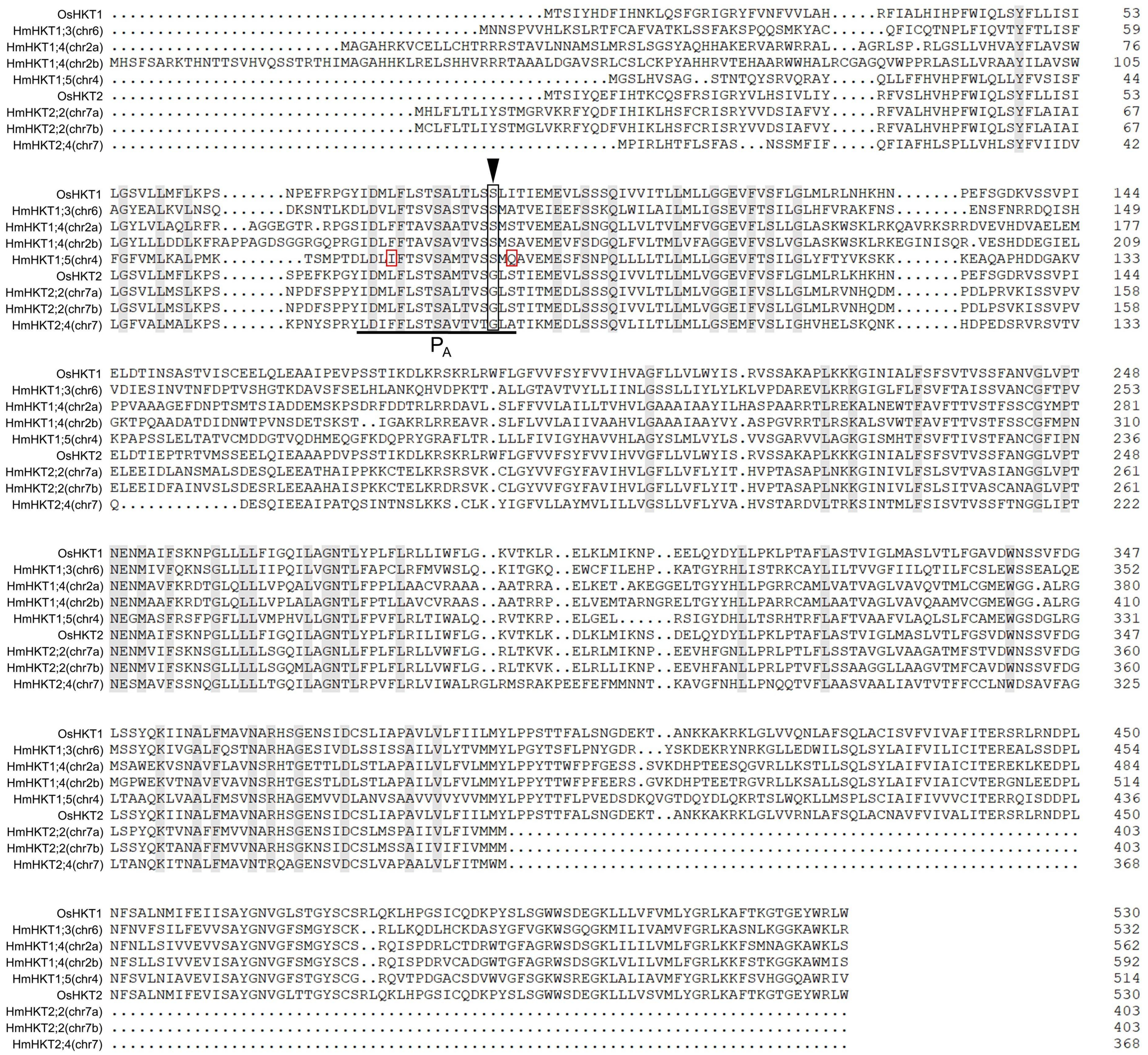

Supplemental Figure 22. Alignment of HKTs amino acid sequences in rice and sea barleygrass.

The conserved serine/glycine residues in the first P-loop (P_A) region are indicated by the black arrowhead and box. Red boxes show the specific residues in HmHKT1;5.

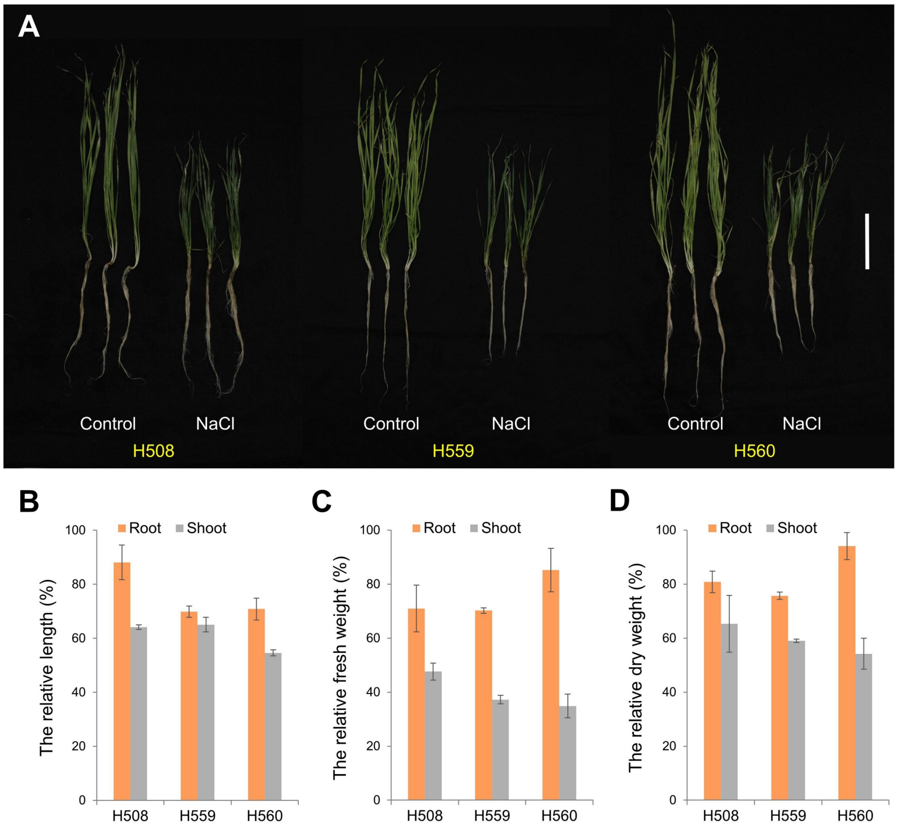

Supplemental Figure 23. The excellent salt tolerance of three spp. *marinum* accessions H508, H559 and H560.

(A-D) The growth performance **(A)**, relative length **(B)**, relative fresh weight **(C)** and relative dry weight **(D)** of roots and shoots of H508, H559 and H560 after treated for one month under 300 mM NaCl. Data are shown as mean ± SD (n=5).

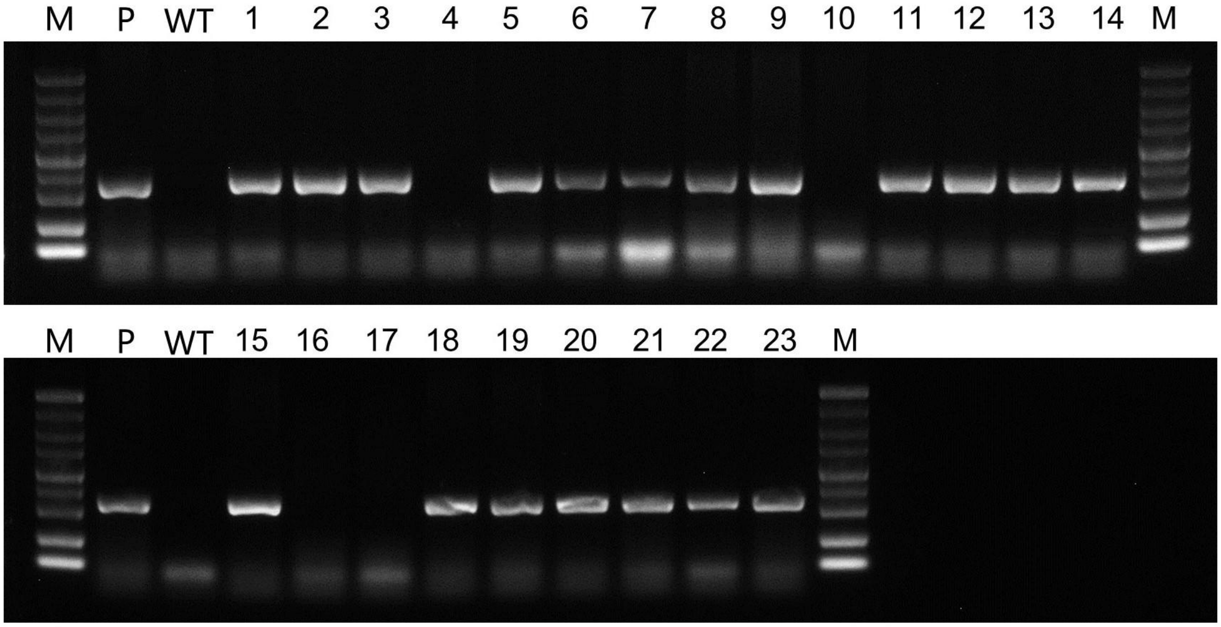

Supplemental Figure 24. The agarose gels illustrating T-DNA insertion in sea barleygrass seedlings detected by the PCR assay.

'P' in the second lane indicates the specific amplicon in the recombinant plasmid.

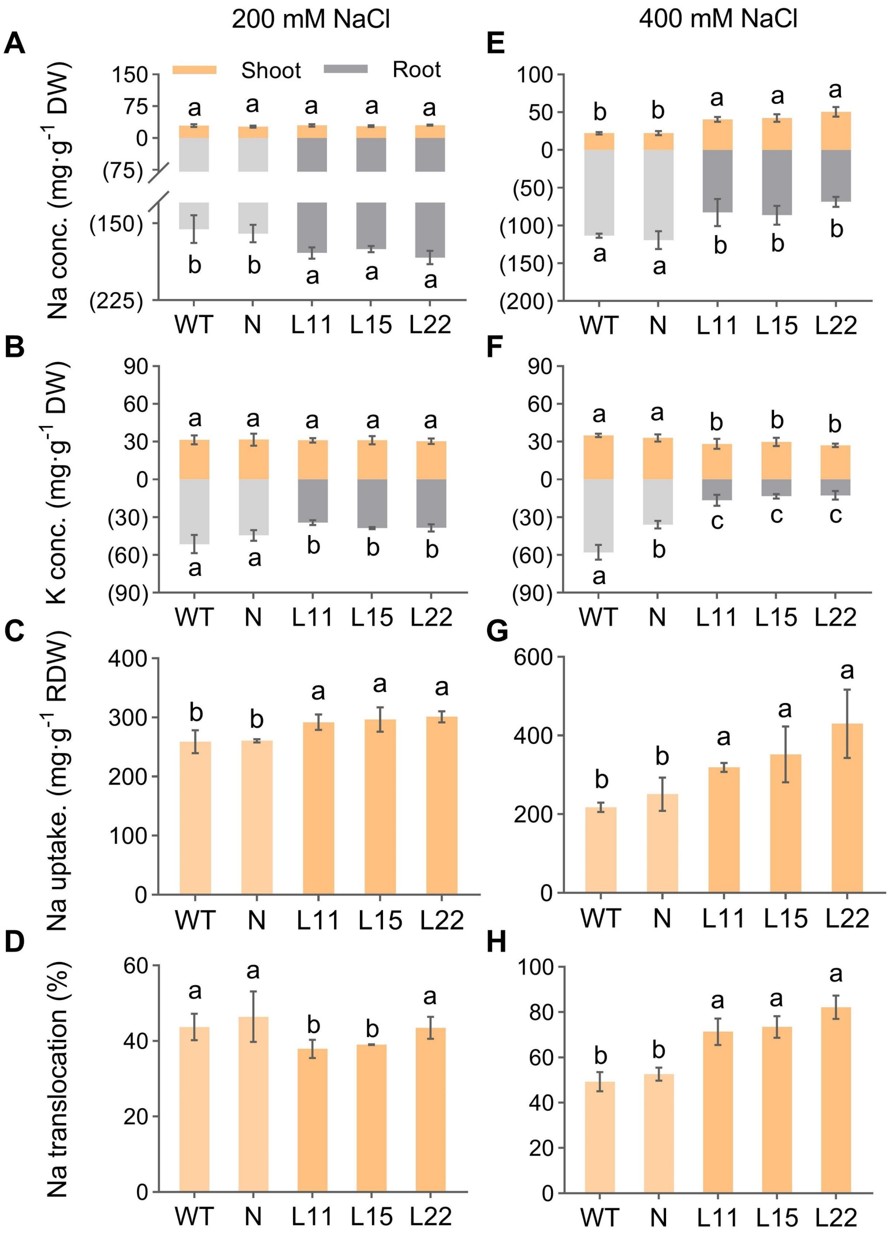

Supplemental Figure 25. The ion content in *hmsos1* mutant and wild-type plants.

(A-D) The Na^+ concentration **(A)**, K^+ concentration **(B)**, root Na^+ uptake **(C)** and Na^+ translocation (%) to shoot **(D)** in *hmsos1* mutants, WT and negative transgenic lines after 14 days of 200 mM NaCl treatment. **(E-H)** The Na^+ concentration **(E)**, K^+ concentration **(F)**, root Na^+ uptake **(G)** and Na^+ translocation (%) to shoot **(H)** in *hmsos1* mutants, WT and negative transgenic lines after 400 mM NaCl treatment for 14 days. Three-week-old seedlings were transferred to hydroponic culture supplemented with 200 and 400 mM NaCl. Root and shoot are shown in grey and brown, respectively. WT, wild type; N, negative transgenic lines without T-DNA insertion. Values are shown as means ± SD (n=4). Different small letters indicate a significant difference ($P < 0.05$) using Tukey's test after a one-way ANOVA.

Supplemental Table 1. *K*-mer analysis predicts characteristics of the sea barleygrass genome.

K-mer	*K*-mer number	*K*-mer depth	Genome size (Mb)	Revised genome size (Mb)	Heterozygous ratio (%)	Repeat (%)
17	192,972,366,592	48	4,020	3,996	0.12	78.45

A total of 266.7 Gb Illumina Hiseq (2×150 bp) data were selected to perform the genome size estimation. Based on the total number of *K*-mer and the corresponding *K*-mer depth of 48, the sea barleygrass genome size was estimated to be 3,996 Mb using the formula: Genome size = *K*-mer_Number/Peak_Depth.

Supplemental Table 2. Statistics of various sequencing data for sea barleygrass genome.

Accession	Illumina (PE 150 bp)		PacBio (Sequel)		10x Illumina		Hi-C Illumina	
	Total data (Gb)	Coverage (×)	Total data (G)	Coverage (×)	Total data (Gb)	Coverage (×)	Total data (Gb)	Coverage (×)
H559	789.1	~207.7	325.3	~85.6	388.4	~102.2	434.9	~114.4

Supplemental Table 3. Statistics on the coverage of reads to the sea barleygrass genome.

Type	Parameter	Percentage %
Reads	Mapping rate (%)[a)]	99.78
Genome	Average sequencing depth[b)]	93.72
	Coverage (%)[c)]	99.84
	Coverage at least 4× (%)[d)]	99.78
	Coverage at least 10× (%)	99.70
	Coverage at least 20× (%)	99.59

[a)]Mapping rate: The ratio of reads to the genome.
[b)]Average sequence depth: The average depth of each base on the genome covered by reads.
[c)]Coverage (%): The ratio of genome covered by reads.
[d)]Coverage at least N×(%): The ratio of genome covered by N× reads.

Supplemental Table 4. Statistics of contigs and genes anchored on seven chromosomes.

Chromosome	Contig number	Length (bp)	HC gene number	LC gene number
Chr1	120	488,453,804	5,293	5,055
Chr2	146	588,092,371	6,773	6,236
Chr3	125	554,163,546	5,897	5,561
Chr4	124	524,471,465	4,722	4,230
Chr5	127	523,182,200	6,262	5,799
Chr6	101	450,127,416	4,758	4,704
Chr7	154	563,686,917	6,276	6,256
Unknown	1,193	121,937,828	1,064	981
Total	2,090	3,815,965,547	41,045	38,822

Supplemental Table 5. The result of BUSCO and LAI assessment on the sea barleygrass genome.

Genome assembly	BUSCO notation assessment results
Hordeum marinum	C:98.4% [S:95.2%, D:3.2%]; F:0.7%; M:0.9%; n:1614[a)]
Hordeum vulgare	C:98.4% [S:95.7%, D:2.7%]; F:0.7%; M:0.9%; n:1614
Triticum aestivum	C:99.0% [S:3.5%, D:95.5%]; F:0.5%; M:0.5%; n:1614

[a)]C: Complete BUSCOs.
S: Complete and single-copy BUSCOs.
D: Complete and duplicated BUSCOs.
F: Fragmented BUSCOs.
M: Missing BUSCOs.
n: Total BUSCO groups searched.

Genome assembly	Total LTR-RTs[a)] length (Mb)	LTR-RTs counts	Complete LTR-RTs length (Mb)	Complete LTR-RTs counts	LAI
Hordeum marinum	2,660.32	106,751	338.46	21,859	12.7
Hordeum vulgare	3,413.62	296,314	398.71	43,957	11.68
Triticum aestivum	8,966.72	450,426	1,354.70	144,385	15.11

[a)]LTR-RTs: LTR retrotransposons

Supplemental Table 6. The comparison of transposable element composition between sea barleygrass and barley genomes.

Classification	Sea barleygrass accession H559		Barley cultivar Morex V3	
	Length (bp)	Ratio (%) in the genome	Length (bp)	Ratio (%) in the genome
Transposable element	3,136.8 Mb	82.20	3,572,6 Mb	84.57
Class I: Retroelement				
LTR Retrotransposons	2,563,853,596	67.19	3,090,790,313	73.17
Copia	1,096,396,193	28.73	962,576,823	22.79
Gypsy	1,377,503,491	36.10	2,080,442,630	49.25
Other	138,533,952	3.63	52,166,036	1.23
Non-LTR Retrotransposons	64,350,220	1.69	65,748,534	1.564
LINE	64,350,220	1.69	24,965,411	0.59
ClassII: DNA Transposons				
DNA Transposons superfamily	428,334,101	11.22	400,773,397	9.49
CACTA superfamily	372,356,638	9.76	332,270,515	7.87
hAT superfamily	3,309,964	0.87	11,543,491	0.27
Mutator superfamily	19,125,361	0.50	16,567,867	0.39
PIF–Harbinger superfamily	10,017,743	0.26	18,293,153	0.43
Tc1/Mariner superfamily	4,409,943	0.12	12,869,676	0.30
MITEs	820,322	0.02	84,485,034	0.02
Helitron	1,094,538	0.03	8,446,967	0.20
Other DNA transposons	104,904,996	2.75	5,530,706	0.13
Other/Unspecified/Unknown	79,007,628	2.07	31,988,909	0.76

Supplemental Table 7. Centromere mapping by *Cereba* and *Quinta* in *H. marinum* genome.

Chromosome	*Cereba* and *Quinta*		
	Median (Mb)	95% CIM[a)] (Mb)	Size of 95% CIM (Mb)
Chr1	137.77	135.37-140.09	4.72
Chr2	250.30	246.22-252.86	6.64
Chr3	217.32	212.15-221.34	9.19
Chr4	223.01	215.65-227.27	11.62
Chr5	151.28	146.89-155.07	8.18
Chr6	218.74	215.47-220.86	5.39
Chr7	301.74	299.09-303.47	4.38

[a)]CIM: Confidence Interval for the Median.

Supplemental Table 8. The gene annotation in protein databases.

	All genes		HC genes	
	Annotated Number	Annotated Percent (%)	Annotated Number	Annotated Percent (%)
NR	63,574	79.6	36,171	88.1
Swiss-Prot	37,297	46.7	24,694	60.2
KEGG	36,579	45.8	23,516	57.3
InterPro	46,562	58.3	27,103	66
Pfam	40,492	50.7	25,264	61.6
GO	32,106	40.2	18,811	45.8
Annotated	64,772	81.1	36,278	88.4
Total	79,867	-	41,045	-

Supplemental Table 9. The statistics of gene structure annotation.

	Hm<br>HC gene	*Hv*<br>HC gene	*Tae*AA<br>HC gene	*Tae*BB<br>HC gene	*Tae*DD<br>HC gene
Number of genes	41,045	35,826	35470	35800	34265
Number of mRNAs	41,045	37,961	43839	44402	42879
Average CDS+intron length (bp)	3,239.46	3060.21	3367.13	3473.29	3394.59
Average CDS length (bp)	1,053.88	1187.45	1309.37	1350.37	1354.05
Average exon length (bp)	255.63	274.80	245.91	254.16	249.62
Average exon number per gene	4.12	4.32	5.32	5.31	5.42
Average intron length (bp)	699.90	563.89	475.83	492.20	461.21

Supplemental Table 10. The statistics of non-coding RNA annotation.

Type		Number	Average length (bp)	Total length (bp)	% of genome
miRNA		19,941	120	2,395,022	0.0628
tRNA		1335	75	99,829	0.0026
rRNA	rRNA	2497	142	354,076	0.0093
	18S	159	564	89,682	0.0024
	28S	161	137	22,027	0.0006
	5.8S	35	144	5,031	0.0001
	5S	2142	111	237,336	0.0062
snRNA	snRNA	921	121	111,220	0.0029
	CD-box	563	102	57,464	0.0015
	HACA-box	150	155	23,250	0.0006
	splicing	208	147	30,506	0.0008

Supplemental Table 11. Genome assemblies used in this study.

Species	Assembly	Data source	Database linkage
O. sativa	IRGSP-1.0	IRGSP	https://rgp.dna.affrc.go.jp/E/IRGSP/download.html
S. bicolor	Sorghum_bicolor_NCBIv3	Joint Genome Institute	https://www.ebi.ac.uk/ena/browser/view/GCA_000003195.3
Z. mays	Zm-B73-REFERENCE-NAM-5.0	NAM genomes	https://download.maizegdb.org/
S. viridis	Setaria_viridis_v2.0	Joint Genome Institute	https://www.ebi.ac.uk/ena/browser/view/GCA_005286985.1
B. distachyon	Brachypodium_distachyon_v3.1	Joint Genome Institute	https://data.jgi.doe.gov/refine-download/phytozome?organism=Bdistachyon&expanded=314
H. vulgare	MorexV3_pseudomolecules_assembly	Leibniz Institute of Plant Genetics and Crop Plant Research	https://www.ebi.ac.uk/ena/browser/view/GCA_904849725.1
T. aestivum	IWGSC RefSeq v2.1	IWGSC	https://wheat-urgi.versailles.inrae.fr/Seq-Repository
H. marinum	This study	--	--

Supplemental Table 12. Single-copy gene families *vs* multiple-copy gene families in 10 Poaceae genomes assessed by OrthoMCL.

Species	Single copy	Percent (%)	2-5 copies	Percent (%)	>5 copies	Percent (%)
B. distachyon	16,427	83.41	3,036	15.42	233	1.18
T. aestivum-AA	18,886	82.46	3,764	16.43	254	1.11
T. aestivum-BB	18,623	81.15	3,859	16.82	316	1.38
T. aestivum-DD	19,136	83.94	3,548	15.56	265	1.16
H. marinum	17,848	83.12	3,311	15.42	314	1.46
H. vulgare	16,200	78.45	4,020	19.47	430	2.08
O. sativa	15,389	82.39	3,128	16.75	163	0.87
S. bicolor	16,896	82.80	3,296	16.15	214	1.05
Z. mays	13,344	69.24	5,578	28.94	351	1.82
S. viridis	16,784	81.26	3,596	17.41	275	1.33

Supplemental Table 13. Ka/Ks values of tillering-, grain size-, and grain shattering-related genes in the genomes of sea barleygrass, barley and wheat.

Gene	*Hm*	*Hv*	*Tae*AA	*Tae*BB	*Tae*DD	*P*-value
Tillering						
Dwarf 3	0.1792	0.1371	0.1737	0.1493	0.1707	1
Dwarf 10	0.1516	0.2157	0.1400	0.1068	0.1407	1
Dwarf 14	0.2093	0.3676	0.2186	0.1866	0.2763	1
IPA1	0.2753	0.3697	0.2809	0.2395	0.2352	1
LAX1	0.1797	0.2736	0.2819	0.2049	0.2274	1
LAX2	0.3386	0.4195	0.2205	0.3849	0.4044	1
MOC1	0.0939	0.1083	0.1151	0.1279	0.1345	1
TB1/FC1	0.2566	0.2779	0.2174	0.2775	0.2593	1
Grain size						
GS1;2	0.0956	0.0887	0.0740	0.0676	0.0650	1
GS1;3	0.1091	0.1130	0.1375	0.1014	0.1354	1
GS2/GL2	0.3679	0.5831	0.3673	0.3401	0.3776	1
GS5	0.1284	0.1448	0.1064	0.1090	0.1285	1
GW5	0.1908	0.1551	0.1587	0.1332	0.1460	0.0040
GW6a	0.3979	0.4100	0.4234	0.4152	0.3707	0.0000
GW7/GL7	0.1536	0.1487	0.1876	0.1826	0.1625	1
GW8	0.2170	0.3724	0.2050	0.1850	0.2267	1
TGW3	0.0411	0.0364	0.0539	0.0386	0.0400	1
TGW6	0.4013	0.3752	0.3278	0.3726	0.3848	0.0016
Grain shattering						
BTR1	0.7025	0.7783	1.3453	0.7335	1.0353	1
BTR2	0.5617	0.4968	0.6263	0.7098	0.5397	1
Q	0.1705	0.1716	0.2090	0.2951	0.2577	1
qSH1	0.3086	0.3066	0.2270	0.2421	0.2481	1
Sh1/SH3	0.0930	0.2115	0.0970	0.0893	0.2617	1
SH5	0.1529	0.1603	0.1397	0.1453	0.1388	1
SHAT1	0.1709	0.1391	0.1072	0.1271	0.1206	0.1704
sh-h/CPL1	0.1180	0.1180	0.0654	0.0809	0.0739	1

The Ka/Ks values of tillering-, grain size-, and grain shattering-related genes among *H. marinum*, *H. vulgare* and *T. aestivum* (AA, BB and DD subgenomes) were calculated by using the branch model of PAML software.

Supplemental Table 14. The amino acid variants of morphology-related genes in sea barleygrass.

Gene	*Hm* (aa)	*Hv* (aa)	*Tae*A/B/D (aa)	Deleterious variant* (*Hv*/*Tae* versus *Hm*)	PROVEAN score
MOC1	426	426	426/429/425	NA	[-0.057, -1.001]
IPA1/SPL17	398	410	410/395/407	S403F	-2.733
BRI1	1126	1118	1120/1124/1121	L778Q	-4.670
				E786D	-2.532
SLN1	618	616	630/631/633	P446S (GRAS domain)	-5.376
BTR1	196	196	194/187/199	NA	[-1.000, 7.000]
BTR2	198	202	198/198/198	D173N	-5.000
VRS1 (Vrs.b2/b3)	221	222	247/217/224	NA	[-1.583, 5.919]
VRS5 (Int-c.b1/b2)	352	355	357/359/357	NA	[-1.000, 7.000]
Nud	227	228	230/228/230	NA	[-1.741, 5.191]

*: The deleterious variants in amino acid sequence are identified by PROVEAN with the score threshold of -2.5.

Supplemental Table 15. The number of salt-tolerant genes in the genomes of rice, sea barleygrass, barley and wheat.

Classification	Gene family	Rice	Sea barleygrass	Barley	Wheat
Proline metabolism	P5CS	2	2	2	6
	ProDH	1	1	1	3
Ion homeostasis	AKT	5	3	5	10
	CHX	28	22	23	62
	CNGC	30	17	17	55
	GLR	24	26	29	77
	HAK	30	28	33	90
	HKT	7	7	6	22
	KAT	4	4	2	9
	KCO	4	3	12	8
	KOR	2	3	2	12
	NHX	6	7	7	18
	PM_ATPase	26	27	31	81
	SOS pathway	3	12	7	27
	TPK	3	3	3	9
	V_ATPase	10	9	11	24
	V_PPase	7	6	13	14
Anti-oxidation	CAT	4	5	5	12
	Cu/Zn SOD	7	5	7	14
	GPX	5	5	5	14
	GST	96	104	110	360
	MnSOD	3	3	3	9
	Peroxidase	177	211	225	641
CBL, CDPK, CIPK	CBL	17	9	7	24
	CDPK	40	41	56	112
	CIPK	41	32	42	96
ABA signaling and synthesis	ABA3	3	4	2	9
	ABI5	3	5	8	29
	CYP707A	2	2	3	6
	NCED5	12	11	14	28
	PP2C	87	70	72	202
	PYL	17	14	13	38
	SAPK	5	9	17	32
	SDIR1	1	3	4	5
	SNRK2	3	3	3	9
	ZEP	1	1	1	3
Methionine metabolism	AMT	14	8	11	21
	AS	8	12	10	28
	GDH	5	4	4	19
	GOGAT	3	2	5	6
	GS	7	7	6	18
	NAR2	2	2	2	8
	NR	3	4	2	9
	NRT	28	41	29	111
	NSR	3	2	9	6
Dehydration tolerance	NIP	12	16	14	35
	PIP	14	18	20	58

SIP	2	2	2	6
TIP	10	11	14	32
Dehydrin	7	8	7	31
ERD	6	10	10	28

Supplemental Table 16. Ka/Ks values of salt-tolerant genes under evolutional selection in the genomes of sea barleygrass, barley and wheat.

Gene/gene family	*Hm*	*Hv*	*Tae*AA	*Tae*BB	*Tae*DD	*P*-value
Ion homeostasis						
AKT1	0.1253	0.1196	0.0920	0.0987	0.0915	0.0494
HAK6	0.2118	0.1695	0.1510	0.1710	0.1693	0.0000
HAK9	0.1207	0.2322	0.1263	0.1014	0.1024	0.0085
HAK23	0.1887	0.1247	0.1234	0.1162	0.1322	0.0000
NHX5	0.2522	0.3617	0.1757	0.1801	0.1801	0.0001
NHX6	0.4056	0.1973	0.1993	0.1801	0.2029	0.0000
CHX15	0.1288	0.1413	0.1062	0.1107	0.0933	0.0006
CNGC4.1	0.1908	0.2645	0.1844	0.1829	0.2118	0.0072
CNGC4.2	0.2537	0.5094	0.2369	0.2368	0.2429	0.0317
CNGC7	0.1391	0.1594	0.1459	0.1609	0.1623	0.0166
GLR2.8	0.4718	0.4192	0.4275	0.5258	0.4766	0.0304
GLR2.9	0.5584	0.5716	0.5366	0.5110	0.4899	0.0001
GLR3.4	0.3002	0.2576	0.2395	0.1950	0.2037	0.0480
HMA4	0.0994	0.0911	0.0660	0.0624	0.0980	0.0000
PM_ATPase 2	0.4240	0.1766	0.1647	0.1472	0.1459	0.0000
PM_ATPase_PAA1	0.3221	0.4285	0.2055	0.2362	0.2350	0.0000
V_ATPase subunit B2	0.0786	0.0631	0.0125	0.0257	0.0248	0.0000
V_ATPase subunit C	0.0702	0.0723	0.0455	0.0469	0.0375	0.0011
V_PPase	0.0671	0.0696	0.0560	0.0841	0.0643	0.0000
V_PPase (OVP1)	0.1100	0.0753	0.0571	0.0552	0.0504	0.0000
Anti-oxidation						
GPX	0.1251	0.0691	0.0436	0.0585	0.0762	0.0015
GST	0.3344	0.2696	0.1830	0.2239	0.1692	0.0000
GST	0.4063	0.4652	0.4464	0.3171	0.4827	0.0000
GST	0.3152	0.3298	0.2858	0.2892	0.3688	0.0000
Peroxidase	0.1675	0.1705	0.1044	0.1080	0.1445	0.0000
Peroxidase	0.2163	0.1961	0.1586	0.1322	0.1167	0.0000
Peroxidase	0.3999	0.3186	0.2773	0.3665	0.3379	0.0000
Peroxidase	0.2017	0.1605	0.1421	0.1319	0.1343	0.0000
Peroxidase	0.3006	0.2464	0.2367	0.1999	0.2430	0.0000
Peroxidase	0.4828	0.3389	0.3643	0.3573	0.3600	0.0000
Peroxidase	0.1845	0.1203	0.1417	0.1632	0.1566	0.0000
Peroxidase	0.3097	0.4096	0.3418	0.2977	0.2424	0.0000
Peroxidase	0.7283	0.5866	0.8466	0.5752	0.5238	0.0001
Peroxidase	0.3377	0.3567	0.4333	0.4569	0.4325	0.0001
Peroxidase	0.2680	0.3374	0.2435	0.3071	0.2479	0.0455
CBL, CIPK, CDPK						
CDPK	0.0182	0.0151	0.0063	0.0168	0.0099	0.0216
CIPK	0.1514	0.1540	0.0925	0.1143	0.1253	0.0000
ABA signaling and synthesis						
CYP707A	0.4776	0.2458	0.2912	0.2681	0.2525	0.0000
NCED5	0.1221	0.1091	0.0897	0.0699	0.0993	0.0000
PP2C	0.3127	0.2247	0.3759	0.3163	0.2333	0.0000
PP2C	0.3551	0.2679	0.2326	0.2509	0.2706	0.0000
PP2C	0.1867	0.1371	0.1119	0.0904	0.1127	0.0000
PP2C	0.4079	0.3875	0.2287	0.2187	0.2138	0.0000
PP2C	0.2103	0.3104	0.1714	0.2273	0.2742	0.0004
PP2C	0.3422	0.2533	0.2578	0.2867	0.2923	0.0152
PP2C	0.3471	0.2179	0.1990	0.2700	0.3005	0.0315

PYL	0.5609	0.3742	0.3779	0.3921	0.3340	0.0001
PYL	0.2385	0.1665	0.2526	0.2559	0.2160	0.0457
SAPK	0.1418	0.1407	0.0184	0.0336	0.0225	0.0000
Methionine metabolism						
AMT	0.1248	0.0923	0.0424	0.0686	0.0463	0.0000
AS	0.1816	0.1702	0.0710	0.0988	0.0930	0.0000
GDH	0.2358	0.1865	0.0560	0.1397	0.1115	0.0000
Dehydration tolerance						
Dehydrin	0.2053	0.2353	0.1379	0.1232	0.0942	0.0000

The Ka/Ks values of salt tolerant- genes among *H. marinum*, *H. vulgare* and *T. aestivum* (AA, BB and DD subgenomes) were calculated by using the branch model of PAML software.

Supplemental Table 17. The list of primers used in this study.

	Gene ID	Forward Primers (5'...3')	Reverse Primers (5'...3')
PCR	*HORMACH01G00325900*	GAGGCTCGCAGTTCCACAAT	CTCACTAGCCGTCGCAACTT
	HORMACH01G00224000	ATCACAGGCAGCAAGAGTCC	CTGCTCACGCATCTGGGATT
	HORMACH02G00830100	GGATCTCCCCTGTTCAACCC	TAACCTCTGCATGTGGGACG
	HORMACH02G00557700	TTGCTGCATCCACTGTGAGT	GCTTACGAGTTAGGACGGCA
	HORMACH03G01487700	TGCTCTGAGTAATGGCCGTC	TATCCCATGGTCGGATTGGT
	HORMACH03G01239700	GATAGCGCCGATGAGGTGTT	GGTGCACGCACATAGTCATC
	HORMACH04G01862100	CCCTGCAGTCACAGAAAATGC	ATGTGCTGGTGTGCGATTCA
	HORMACH04G01978300	TACTCTCCACGAGGCCGTAA	GTCCAGTTGCAGCCTCTCAT
	HORMACH05G02549700	CCACTCGCATTCTCCAGGAC	TCTGAGAGAAGAGCCACGGT
	HORMACH05G02635100	CGACAGCTGAAGTGGACCTG	GTCTCCAGGCGAAGGTTCTG
	HORMACH06G03023500	ACATCGCTGCATATGGCACT	GCAGCAAAGGCAACAGTCAT
	HORMACH06G03353900	AGGGAGTACCAAATGCAGCG	ACGCACGACGGACTAGATTG
	HORMACH07G03592100	CACGGAAGTATAGCCGTCCC	CACTGATGGCTACCGATCCC
	HORMACH07G03703600	TGTTTGCAAGGATTCCGGGT	GCGGTGGCACAAACATAGAC
RT-qPCR	*HmActin*	GACTCCGGAGACGGTGTCAGC	GGCTGGAAGAGGACCTCAGG
	HvActin	GACTCTGGTGATGGTGTCAGC	GGCTGGAAGAGGACCTCAGG
	TaActin	CCTCTCTGCGCCAATCGT	TCAGCCGAGCGGGAAATTGT
	HORVU5Hr1G105840	GTTTGGTGATGTAAACGCTCTT	AACAGGTGCAACAGTTTAACTC
	HORVU6Hr1G008640	GGATCAAGAAGGAGAACGACTT	GGAAGTCGATCCAGATGACAC
	HORVU2Hr1G110230	GATACTCAGCGTACAGTACTCC	GCCGTGACAAGAATATACAACC
	HORVU2Hr1G045200	GCACGTGATTGAAAACCTAACT	GACTAGCGTACTACTCCTATGC
	HORVU2Hr1G113070	CTTACACACTACGGACTGAAGT	CATGCTTGGATGATGTCATTGA
	HORVU5Hr1G008270	GAAGCTCCTCACCACTGACATC	CCATCTCCAGAGAGTGCAAC
	HORVU1Hr1G053440	GGATCGGCGAGCGTGTGTAT	GACGGGTTCTTGTGCTGCCA
	HORVU1Hr1G005800	GGCCTCGCCTTCGCCCTCAA	CCACTCCGGAAAGAGTGTGC
	HORVU6Hr1G031360	TGGAGTTCCGAGGCTTTGCAG	CACCAGCATGCCTCGCATTG
	HORVU3Hr1G003150	GTGCAAGCTGCTTACTGGGGA	TGGACCGCAAACCCTTCCAA
	HORMACHUnG03807500	CGCCTTGGCCCAAACTACCT	TTTCCGACAACGGGTCGAGT
	HORMACH06G02877200	TCCAGCTCCAGCTCGTCTGA	GTCCTTGTGGGCTCCGCC
	HORMACH01G00260100	CCAGGTGGGAGGAGCGGTTC	ACTTGGCCTTGGAGGCGATGA
	HORMACH07G02961700	CTCGGCGTGCTATCGTTGGT	ACGTGCCACCTTCTCCGTTG
	HORMACH04G01818300	GCGCACCATTGTTGTCCTCT	GGCACGAGCGGGAGTAGAAA
	HORMACH03G01265700	CACCGCAGTCGAGCAAAACA	CAACCATGTCAACGTTCCCGTC
	HORMACH05G02491500	GCACCTGCATCCCAAACCGA	CCGCGGCGAGGTTTAAATGC
	HORMACH02G00961900	CAATCCAGCTGCGTTCAGCG	GAGCCAGTCAACGGGCTGAG
	HORMACH01G00199500	GGCATCGCCTTCGCTCTCAA	CCACTCCGGAAAGAGGGTGC
	HORMACHUnG04050000	GTGCAAGCTGCTTACTGGGGA	TGGACCGCAAACCCTTCCAA
	TraesCS6A01G041700	GATCTTGCTGGAGGACTACC	GTGGATCACCGTGGAAAAG
	TraesCS1A01G186100	CATTCTGCAACCACATTCATCA	AAGGCTCTCCTCCACGTA
	TraesCS4B01G059100	CCAAGATGGCGAATCATTACAC	ATTGCTAATTCTTCGATTCCGC
	TraesCS3B01G234100	GAACAAGAAAGCACTACACAGG	CGATGTACTCGTAGCTGAGG
	TraesCS3D01G518200	GACAACTCCACCGCCAACGA	CTGGTGACCACGGGCTTTGT
	TraesCS3D01G355900	GCCGTTGCCTCTTCGGTGAT	CGTACCTCGGGCAACGATCC
	TraesCS7A01G331300	ATGGATGTTGTGAAGTTGTTGG	TGGAGGTATGTTAAACGACACA
	TraesCS6B01G364600	CACGGGGGTGTACAAGAAG	GAGATCAGGAGGAGATCAGC
	TraesCS1A01G037400	AACCTCATCGAGACCATCCG	GTAGGTGCCGAAGGCGAC
	TraesCS6D01G144500	GGCAATTTTAATGCTGATTGGC	CAGGCGGATTTCAGAAAATGAA
	HmSOS1	CCACACGGCGTTTGCTCTCT	TCGTCGCTCGAGTTGCCATC
	HvSOS1	ATCAGTGGTGCATTGTTTCTTC	CAATAACGAGAGCACTTTCCTG
	HmHKT1;5_P1	CCTCCTGGGAAACACGCTCT	GTCAGGAGGTGGTCGTAGCC
	HmHKT1;5_P2	TGGAGCAAGGGTTCAAGGAC	AGGTAGACCAGCATCAGGGA
	HvHKT1;5_P1	CCTCCTCGGAAACACGCTCT	GTCAGGAGGTGGTCGTAGCC
	HvHKT1;5_P2	TGGAGCAAGGGTTCAAGGAC	AGGTAGACCAGCATCAGGGA

Absolute quantification	*SOS1*	GTGCAAGCTGCTTACTGGGGA	TGGACCGCAAACCCTTCCAA
	HKT1;5	CCGTCGAGGTTATCAGTGCGT	CATGACGGCTATGAGGGCGA
Vector construction	*HmSOS1*	CTTGAAGACGGCCAGGAGGAGG T	AAACACCTCCTCCTGGCCGTCTT
T-DNA insertion	*Cas9*	CCTGGCCCACATGATCAAGT	TGTACTTCTCAGGCAGCTGC
PCR-RE	*HmSOS1*	TTCATCTGTGGTGCTTCACGTA	TGACAGACAGAAGGAAGAAGGAG
Sanger sequencing	*HmSOS1*	CCGGCATCCGCATCTGTA	CCGGAAATACAAACTATTCCAGGC

Other Supplemental information for this manuscript includes the following:
Supplemental Dataset 1. The genes involved in the expanded, contracted and species-specific gene families in the sea barleygrass genome.
Supplemental Dataset 2. The identified deleterious amino acid variants in *H. marinum*.
Supplemental Dataset 3. The list of the accession numbers/gene IDs used in this study.
Supplemental Dataset 4. The data quality and information of the RNA-seq libraries.
Supplemental Dataset 5. The expression and detailed information of the DEGs.
These Datasets are available online as an Excel file.
